# Mass spectrometry-based metabolomics approach in the isolation of bioactive natural products

**DOI:** 10.1038/s41598-020-58046-y

**Published:** 2020-01-23

**Authors:** Daniel P. Demarque, Renata G. Dusi, Francisco D. M. de Sousa, Sophia M. Grossi, Maira R. S. Silvério, Norberto P. Lopes, Laila S. Espindola

**Affiliations:** 1 Laboratório de Farmacognosia, Universidade de Brasília, Brasília, Brazil; 2 Núcleo de Pesquisa em Produtos Naturais e Sintéticos, Departamento de Física e Química, Faculdade de Ciências Farmacêuticas de Ribeirão Preto, Universidade de São Paulo, Ribeirão Preto, Brazil

**Keywords:** Metabolomics, Target identification, Secondary metabolism

## Abstract

Metabolomics is a powerful tool in the analysis and identification of metabolites responsible for biological properties. Regarding natural product chemistry, it constitutes a potential strategy to streamline the classic and laborious process of isolating natural products, which often involves the re-isolation and identification of known compounds. In this contribution, we establish a mass spectrometry-based metabolomics strategy to discover compounds with larvicidal activity against *Aedes aegypti*. We analyse the Brazilian plant *Annona crassiflora* using different platforms to annotate the active compounds in different extracts/fractions of various plant parts. The MetaboAnalyst and GNPS platforms, which consider LC-MS and LC-MS/MS data, respectively, were chosen to identify compounds that differentiate active and inactive samples. Bio-guided isolation was subsequently performed to confirm compound activity. Results proved the capacity of metabolomics to predict metabolite differences between active and inactive samples using LC-MS and LC-MS/MS data. Moreover, we discuss the limitations, possibilities, and strategies to have a broad view of vast data.

## Introduction

Discovery and characterization of chemicals from natural product (NP) sources has inspired the development of many products and medicines, encouraging many research groups to dedicate efforts to sourcing molecules from plants, bacteria, fungi and other natural origins. The classical approach in the discovery of bioactive NP typically starts with biological screening of crude extracts, followed by fractionation procedures until the isolation and identification of the bioactive compound(s)^1–3^. Although successful, the classical tools that have led to the discovery of chemical entities are time consuming and often inefficient to discover new compounds. Patridge and co-workers^4^ analyzed FDA-approved drugs from natural sources and observed a declining trend in the contribution of natural products to new molecular entities, especially from plant sources. As the biodiversity remains largely unexplored, the classical NP isolation process often involves the isolation of known compounds^5,6^. To overcome this discovery bottleneck, the association of analytical tools with computational and statistical treatments, known as “omics” tools, constitutes a powerful ally for natural product chemists.

Metabolomics, which refers to the identification and/or quantification of small molecules produced by a biological system at a specific point in time analyzable by the chosen technique, can facilitate and accelerate the search for novel active agents. The association of metabolomics with statistical methods provides a better view of vast data and simplifies the analysis required to answer the question posed. In this context, mass spectrometry (MS) and Nuclear Magnetic Resonance (NMR) are the most commonly employed analytical techniques in metabolomics analysis. The advantages of MS compared with NMR are: sensitivity, small sample volume and the possibility of coupling with a chromatographic technique^7^. Moreover, MS/MS fragmentation data provides additional useful information for structural elucidation and comparison with databanks^8^.

In this contribution, we applied mass spectrometry-based metabolomics and chemometric tools to source natural bioactive compounds. We used *Annona crassiflora* extracts from different plant parts to develop a model to improve the discovery of larvicidal compounds against *Aedes aegypti* within the ArboControl Brasil Project. Moreover, we validated the model by using a traditional bio-guided isolation approach and discussed both the limitations and possibilities of different approaches for the isolation of bioactive natural products.

## Results

The initial motivation of this work was the presence of 195 crude extracts active against *Ae. aegypti* larvae (data not shown) detected in previous high-throughput screening (851 samples) of the *Brazilian Cerrado biome Plant Extract Bank* (Laboratório de Farmacognosia/Universidade de Brasília). In order to tackle this vast number of active extracts and define the compounds of interest prior to isolation, we developed the approach presented herein. *Annona crassiflora* was chosen to develop this model using hexane and ethanol crude extracts from different plant parts (stem wood - SW; leaves - L; root bark - RB; root wood – RW, and stem bark - SB). Prior to metabolomics analysis, crude extracts were partitioned to clean up fractions and increase chemical profile variability.

### Larvicidal and HPLC-DAD-MS/MS analysis of fractions from different *Annona crassiflora* extracts

Eight *A. crassiflora* crude extracts were partitioned with Diol cartridges using hexane (Hx; clean-up), ethyl acetate (EtOAc) and methanol (MeOH) as solvents. In the present study, only the EtOAc and MeOH phases were used due to the solubility in water required for the larvicidal tests and suitability to perform HPLC-DAD-MS/MS analysis. Fractions were subsequently dried and those with a minimum of 5 mg yield (13 fractions; 7 EtOAc and 6 MeOH) were submitted to biological tests and HPLC-DAD-MS/MS analysis. The latter were performed for active and inactive samples using positive ion acquisition mode (see Tables S1 and S2 for yields and larvicidal activity). Fractions were considered active when larvae mortality was higher than 10% at 125 µg/ml.

**Figure 1:**
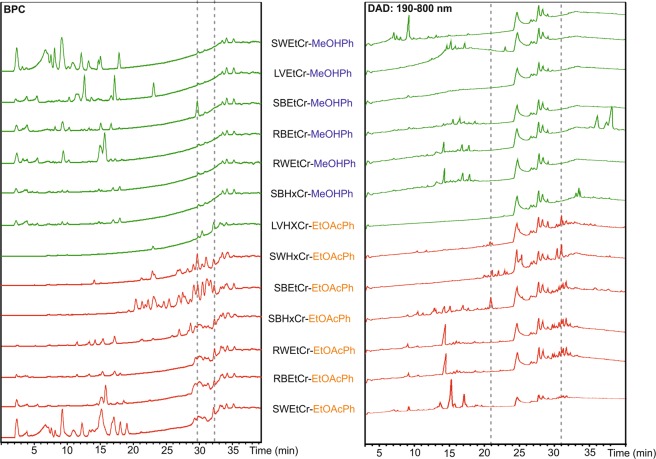
Chromatograms: BPC - base peak chromatogram and DAD - diode array detector, of active (red) and inactive (green) fractions obtained from crude extracts. SW: stem wood; L: leaves; RB: root bark; RW: root wood; SB: stem bark; EtCr: ethanolic crude extract; HxCr: hexanic crude extract; MeOHPh: methanolic phase; EtOAc: ethyl acetate phase.

Six of the 7 EtOAc fractions were active, while none of the methanol fractions presented activity. Normally, partitioning results in different chemical profiles. However, chromatogram analysis (base peak chromatogram – BPC and diode array detector – DAD) in Fig. 1 shows that the chemical profiles remain complex. This strategy is effective to unbalance the chemical profiles and could be especially useful if several extracts from the same species are unavailable.

Visual inspection can give some indications, for instance, the BPC shows compounds with a retention time (RT) of ~30 min that are present mostly in active fractions, although some of them are also present in the inactive fractions (Fig. 1). Moreover, the UV chromatogram (DAD) shows the presence of compounds in the 21–23 min range which are only present in active samples, despite some of them being at a low concentration. In addition, compounds with an RT 31 min are mostly present in active samples, except in SWEtCr-EtOAcPh, in which the most intense compounds are those present in the ~15 min range. Despite these differences and there being some indications, it is still not possible to use visual inspection to accurately determine which compounds are present in active fractions and/or missing (or reduced) in inactive fractions. This procedure, without considering the level of these compounds in different samples, may result in an incorrect interpretation. Additionally, in a situation with limited sample availability for manual inspection, it is important to note that, even if the difference is clearly observable, the co-eluted compounds would not be discernable.

Considering that visual inspection alone is often difficult to accurately determine the active compounds, not to mention impractical for multiple samples, untargeted metabolomics and statistical analysis were performed in 2 different platforms: MetaboAnalyst (LC-MS data) and GNPS (LC-MS/MS data).

### MetaboAnalyst platform

**Figure 2:**
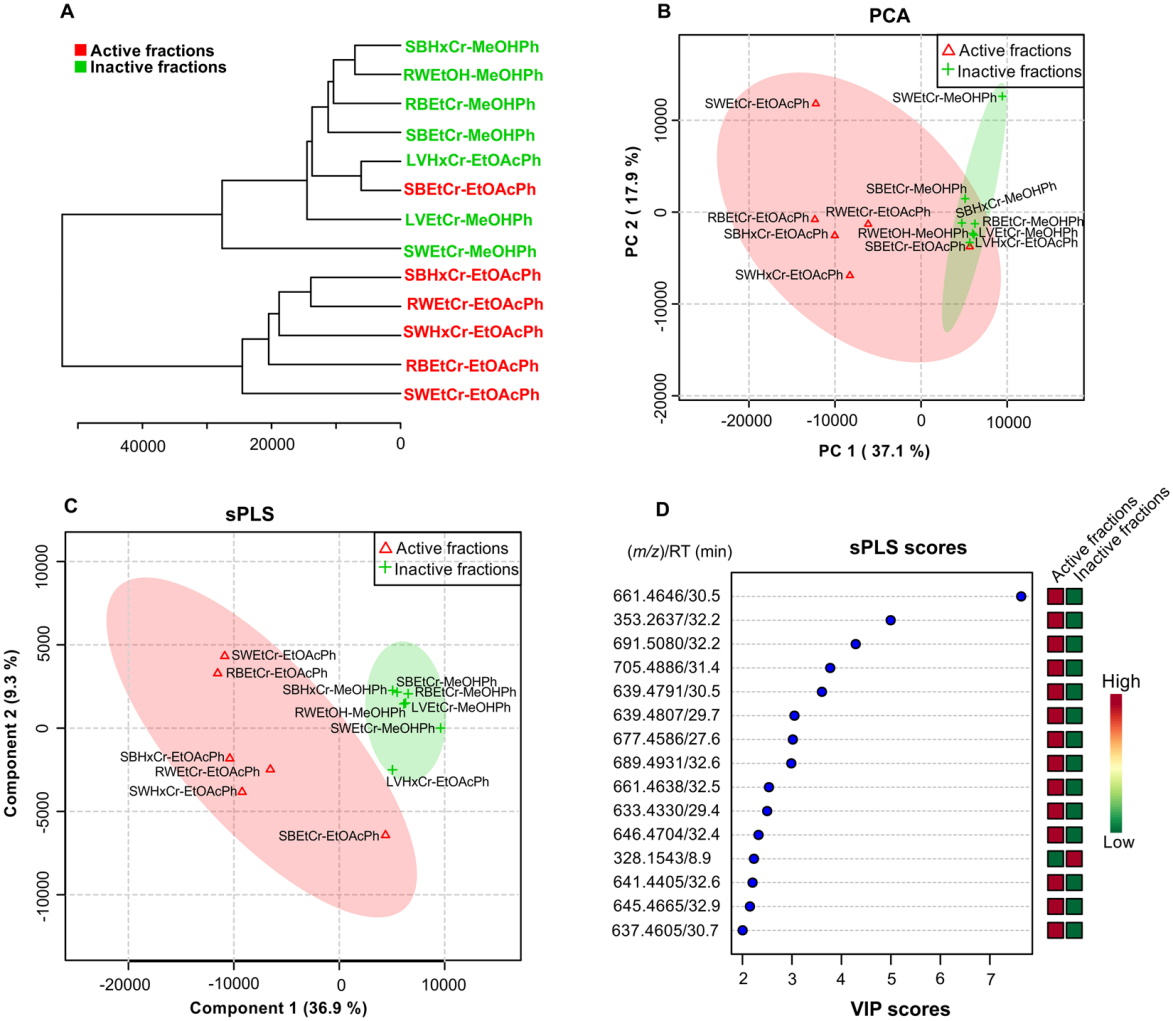
(**A**) HCA and (**B**) PCA plots of unsupervised methods; (**C**) sPLS plot and (**D**) sPLS loadings for the 25 most important variables. SW: stem wood; L: leaves; RB: root bark; RW: root wood; SB: stem bark; EtCr: ethanolic crude extract; HxCr: hexanic crude extract; MeOHPh: methanolic phase; EtOAcPh: ethyl acetate phase.

The first platform used to detect differences between active and inactive samples was MetaboAnalyst^9^. This exploratory statistical analysis platform considers the retention time and molecular weight of compounds (LC-MS data). The input file is a table with feature (*m/z*), sample name, group (active/inactive) and the area of each peak. To generate this table, LC-MS/MS data were converted to.mzXML format using MSConvert software. The.mzXML files were pre-processed in MzMine to construct chromatograms from the detected *m/z* masses, eliminating baseline interference and isotopes (see methods for details). After pre-processing, the chromatograms were aligned, built considering MS1 data only and exported as comma-separated values (.csv). This file was uploaded to MetaboAnalyst with the results presented in Fig. 2. Statistical analysis was performed using unsupervised (hierarchical cluster analysis – HCA, and principal components analysis – PCA) and supervised (partial least squares projection to latent structures – sPLS) methods.

The HCA plot clearly segregates 2 groups: one containing all active fractions and the other grouping the inactive fractions, with the exception of one active sample. In the PCA plot, 2 components account for 55% of the variance. Active fractions were mainly restricted to a different area, despite being inside the confidence region of inactive fractions. These 2 unsupervised methods point towards statistically significant differences between the 2 groups. In the supervised method sPLS, taking into account the 15 most important variables to differentiate between the 2 groups, the separation was clearly visualized in the plot (Fig. 2D). Of the 15 most important features for group separation, we verified that: *m/z* 353.2637, *m/z* 328.1543, *m/z* 646.4704 and *m/z* 645.4665 were source fragment, noise and isotopes, respectively. After changing several parameters in MzMine, we noticed that these peaks can still appear in the analysis under close inspection. From the remaining 11 compounds, we annonated 10 known Annonaceous acetogenins corresponding to the following molecular formulas: C_37_H_66_O_8_ + Na^+^ (661.4646, error 0.4 ppm, RT 30.5 min); C_35_H_68_O_8_ + Na^+^ (639.4791, error 3.2 ppm, RT 30.5 min); C_35_H_68_O_8_ + Na^+^ (639.4807, error 0.7 ppm, RT 29.7 min); C_37_H_66_O_9_ + Na^+^ (677.4586, error 2.7 ppm, RT 27.6 min); C_35_H_62_O_8_ + Na^+^ (633.4330, error 1.9 ppm, RT 29.4 min); C_37_H_62_O_7_ + Na^+^ (641.4405, error 1.9 ppm, RT 32.6 min); C_35_H_66_O_8_ + Na^+^ (677.4586, error 7.9 ppm, RT 30.7 min); C_39_H_72_O_8_ + Na^+^ (691.5080, error 6.5 ppm, RT 32.2 min); C_39_H_70_O_9_ + Na^+^ (705.4886, error 4.4 ppm, RT 31.4 min), and C_39_H_70_O_8_ + Na^+^ (689.4931, error 5.4 ppm, RT 32.6 min). The compound *m/z* 667.4638 was not identified. We also observed a predominance of these active compounds with retention times between 29–32 min.

### GNPS platform

**Figure 3:**
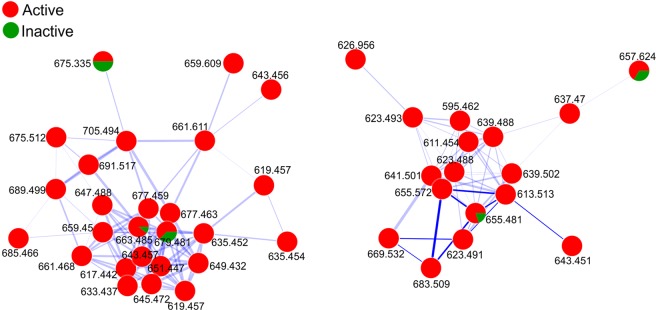
The 2 main clusters of active compounds from molecular networking.

The LC-MS/MS converted data (.mzXML) files were uploaded to the global natural products social molecular networking (GNPS) platform which organizes vast mass spectrometry datasets according to similarity between fragmentation patterns (MS/MS) of related precursor ions. Closely-related compounds (with similar fragmentation profiles) are grouped in clusters rendering data mining less arduous while providing clearer data visualization. Profound investigation of the clusters highlights analogous molecules and, as it compares MS/MS spectra with robust databases, facilitates the dereplication of compounds and/or classes of compounds. Moreover, each node is a pie chart showing compound distribution in different groups (in this study: active/inactive). The networking in the present study found clusters relating to compounds that are mainly present in active samples (red) Fig. 3 (complete networking is presented in Fig. S1). According to the *m/z* obtained in the nodes (precursor ion, MS1), the corresponding molecular formulas were calculated considering a maximum 7 ppm error. The annotated compounds are presented in Table 1; and are attributed to acetogenin derivatives, which typically contain between 35 and 39 carbons.

**Table 1:** Calculated molecular formula of compounds reported in Clusters 1 and 2.

*m/z*	Formula (error)
**Cluster 1**	
643.456	C_37_H_64_O_7_ + Na^+^ (1.6 ppm)
677.463	C_37_H_66_O_9_ + Na^+^ (3.8 ppm)
633.437	C_35_H_62_O_8_ + Na^+^ (4.4 ppm)
617.442	C_35_H_62_O_7_ + Na^+^ (4.4 ppm)
661.468	C_37_H_66_O_8_ + Na^+^ (3.8 ppm)
645.472	C_37_H_66_O_7_ + Na^+^ (2.2 ppm)
659.450	C_37_H_64_O_8_ + Na^+^ (0.2 ppm)
619.457	C_35_H_64_O_7_ + Na^+^ (3.3 ppm)
649.432	C_35_H_62_O_9_ + Na^+^ (4.4 ppm)
663.485	C_37_H_68_O_8_ + Na^+^ (5.8 ppm)
647.488	C_37_H_68_O_7_ + Na^+^ (2.7 ppm)
651.447	C_35_H_64_O_9_ + Na^+^ (3.4 ppm)
635.452	C_35_H_64_O_8_ + Na^+^ (3.4 ppm)
689.499	C_39_H_70_O_8_ + Na^+^ (3.2 ppm)
685.466	C_39_H_66_O_8_ + Na^+^ (0.7 ppm)
**Cluster 2**	
623.493	C_37_H_66_O_7_ + H^+^ (7.0 ppm)
639.488	C_37_H_66_O_8_ + H^+^ (6.9 ppm)
611.454	C_35_H_62_O_8_ + H^+^ (2.8 ppm)
623.488	C_37_H_66_O_7_ + H^+^ (1.0 ppm)
641.501	C_37_H_68_O_8_ + H^+^ (2.8 ppm)
655.481	C_37_H_66_O_9_ + H^+^ (3.8 ppm)
669.532	C_37_H_74_O_8_ + Na^+^ (5.8 ppm)
683.509	C_37_H_72_O_9_ + Na^+^ (2.4 ppm)

### Bioactivity-guided isolation of active compounds

The use of metabolomics highlighted the presence of bioactive acetogenins in active fractions. To confirm that acetogenins were responsible for activity, bioassay-guided isolation of active compounds was performed with subsequent identification. Classical methodology was employed, testing all fractions at each stage, choosing the active one(s) to proceed to the next purification step. This methodology, without considering the results found in metabolomics analysis, was chosen to verify if both the metabolomics and bioassay-guided isolation approaches would ultimately point to the same active compound(s).

The active *A. crassiflora* stem wood hexanic extract (8.7 g) was partitioned in a silica cartridge (174 g) and eluted with 800 ml of hexane (yield 10%), ethyl acetate (yield 21%) and methanol (yield 69%). The larvicidal activity of the resulting fractions was tested at 125 µg/ml, and ethyl acetate was active with 92.5% mortality. The ethyl acetate fraction was purified in a silica gel chromatography column, yielding 7 fractions. Subsequent larvicidal assays revealed 1 active fraction, which was further chromatographed in a Sephadex LH-20 column, resulting in 6 fractions of which the bioactive one was further purified by preparative-HPLC. Due to the high complexity of the chemical profiles and poor chromatographic resolution, we obtained 4 fractions, which were ultimately identified as mixtures by NMR and MS. The active compounds were identified as Annonaceous acetogenins, polyketide-derived fatty acid derivatives usually containing between 35 and 39 carbons. These compounds are characterized by tetrahydrofuran rings, a single methylated gamma-lactone moiety and several hydroxy, acetoxy, and/or ketone groups along the hydrocarbon chain^10^.

In fraction Prep_Fr1 we identified 2 Annonaceous acetogenins. Direct-infusion MS showed ions compatible with molecular formulas of C_37_H_66_O_8_ + Na^+^ (m/z 661.4363, error 2.9 ppm) and C_37_H_66_O_9_ + Na^+^ (m/z 677.4570, error 5.1 ppm). Fraction Prep_Fr2 contained a mixture of: C_37_H_66_O_8_ + Na^+^ (m/z 661.4631, error 3.6 ppm); C_35_H_64_O_8_ + Na^+^ (m/z 635.4476, 3.6 ppm), and C_37_H_68_O_9_ + Na^+^ (m/z 679.4760, 0.1 ppm). Fraction Prep_Fr3 consisted of: C_35_H_64_O_8_ + Na^+^ (m/z 635.4493, 0.9 ppm); C_37_H_66_O_7_ + Na^+^ (m/z 645.4695, 1.7 ppm); C_35_H_62_O_7_ + Na^+^ (m/z 617.4386, 1.1 ppm); C_37_H_68_O_8_ + Na^+^ (m/z 663.4792, 3.0 ppm), and C_39_H_70_O_9_ + Na^+^ (m/z 705.4895, 3.2 ppm). Fraction Prep_Fr4 corresponded to C_37_H_68_O_8_ + Na^+^ (m/z 663.4788, 3.6 ppm). When submitted to collisional activation, these molecules showed typical fragmentation from dehydration reactions (−18 Da) and loss of the lactone ring (−112 Da) (Figs. S3–S15)^11^.

**Figure 4:**
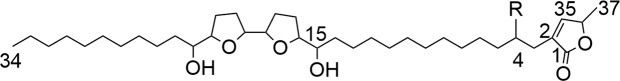
An example of acetogenin chemical structure. (R = H, subtype 1a annonacin; R = OH, subtype 1b squamocin).

Acetogenin identification was corroborated with NMR analyses, where signals from α,β-unsaturated-γ-lactones which characterize the vast majority of Annonaceous acetogenins were present in the NMR spectra of all Prep fractions (Figs. S16–S34). The chemical shift of the lactone ethylenic proton distinguishes the subtype visualised in each fraction: protons at around 6.94 ppm are typical of subtype 1a annonacin-like Annonaceous acetogenins (Fig. 4), while more upfield 7.2 ppm protons are found in subtype 1b squamocin-like Annonaceous acetogenins (C-4 hydroxylated)^12^. A mixture of both subtypes was detected in Prep_Fr1 and Prep_Fr2, while only type 1a was present in other fractions. Deshielded oxygenated ^13^C chemical shifts confirmed the presence of tetrahydrofuran moieties and hydroxyl groups, however it was not possible to determine their exact location in the long alkyl chain as they were observed as overlapping downfield signals in the spectra. After identification, the median lethal dose (LD_50_) of each fraction was determined. All fractions were active and the LD_50_ ranged from 5.6–11.1 µg/ml (graphics Fig. S35–S38).

## Discussion

Traditional natural products chemistry relies on a biological-guided isolation approach for drug discovery. The metabolomics strategy aims to skip the isolation step by exploiting computational and statistical tools to directly compare groups, identify the most significant features and, therefore, the active compounds^13^.

The existence of differences between the chemical profiles of samples was determined by an untargeted metabolomics approach. Targeted metabolomics is based on the quantitative measurement of known compound concentrations, whereas untargeted metabolomics involves high-throughput measurement of all compounds analyzable by the chosen technique. As a broad analysis, the vast amount of data generated by LC-MS/MS renders manual interpretation unfeasible. Multivariate analysis or molecular networking can organize the data, enabling the detection of patterns and differences between groups as it considers all metabolomic features. Unsupervised and supervised methods can be adopted for pattern recognition in multivariate analysis. In the former, all metabolomic features are considered without previous recognition of different groups, while the latter considers and maximizes features to distinguish the predefined groups (e.g. active and inactive)^14^. The GNPS platform uses fragmentation patterns to group compounds, thereby organizing visualization and permitting comparison with databases to facilitate the dereplication process^8^.

In the present study, we used 2 methods to analyze LC-MS and LC-MS/MS data. As MetaboAnalyst does not consider fragmentation, even peaks that did not achieve the minimum intensity to perform the fragmentation (depending on the acquisition settings) could appear in the analysis. One alternative to overcome this issue is to select higher intensities in the chromatogram builder during data pre-treatment. However, many other features can still be detected such as in-source fragments and impurities, which can complicate the dereplication process. Even if it is not possible to identify the active compounds using the MetaboAnalyst platform, these results still provide useful information in terms of retention time and can guide the analyst where to inspect (in the chromatogram) in order to find the target compound. The predominant retention time of compounds that differentiate the groups are present in the 29–32 min range (Fig. 2D).

An advantage of using MetaboAnalyst is the “Variable Importance in Projection” (VIP) score in the sPLS plot, which indicates the most important compounds that differentiate the groups. The most important compound present in the active samples indicated by VIP of sPLS was *m/z* 661.4646 (C_37_H_66_O_8_ + Na^+^, RT 30.5 min), which was isolated using a bio-guided approach, together with 2 other compounds indicated by MetaboAnalyst. Great care must be taken when analyzing the data, with consideration given to the chosen parameters, as baseline, isotope and other in-source fragment peaks can appear. Regarding other compounds VIP scores, as mass spectrometry is a very sensitive technique, more compounds can be annotated in comparison with classical bioassay-guided fractionation. Additionally, we showed that this methodology is applicable, even for such complex chemical profiles, although more attention must be given during data analysis. The acquisition of only LC-MS data (without fragmentation) could improve data for MetaboAnalyst platform analysis as it does not spend time in MS/MS scanning. However, we showed that it is possible to leverage the LC-MS/MS data for use in both the GNPS and MetaboAnalyst platforms, thus avoiding the need for 2 separately acquisitions of the same sample.

In the GNPS platform, clusters are formed considering similar fragmentation patterns. Therefore, the absence of a class of structurally-related active compounds could hinder the dereplication process. In this case, it could result in nodes that do not link to other groups (self-loop)^15^. Moreover, even when no hits are found in the databank, the organization in clusters (with similar fragmentation profiles) performed by GNPS facilitates analyzing the results and dereplication process.

Five of the isolated compounds were among those annotated in GNPS analysis. While GNPS indicates the relative concentration of the compound in different groups (as represented in the pie chart), it is the VIP score in the sPLS plot (MetaboAnalyst) which indicates the most important compounds that differentiate the groups and could be responsible for the activity. In the case presented in this study, the high complex chemical profile with several active compounds can complicate data analysis, therefore, we consider both strategies valuable to understand vast data.

The metabolomics approach pointed to Annonaceous acetogenins as the compounds responsible for differentiation between active and inactive samples. These results were subsequently validated by bioassay-guided fractionation. The aforementioned compounds were previously identified as larvicidal agents, with known mechanisms of action in the larvae anal papillae^16–18^. Efforts to mine molecules from this species without a metabolomics approach could result in the isolation of known compounds, with no innovative result. Metabolomics can avoid this or, when this is the goal, highlight the target. Furthermore, knowledge of physicochemical properties and/or previous purification procedures facilitates the isolation process. Other advantages include prevention of sample degradation and loss of low concentration compounds during the isolation process, not to mention the avoidance of time and cost-consuming methodologies when the intention is to obtain novel compounds. Nevertheless, it was possible to annotate other Annonaceous acetogenin derivatives by MS that would be difficult to obtain using the classical isolation approach due to low quantities. The results found in this contribution therefore support the routine use of metabolomics in NP chemistry.

**Figure 5:**
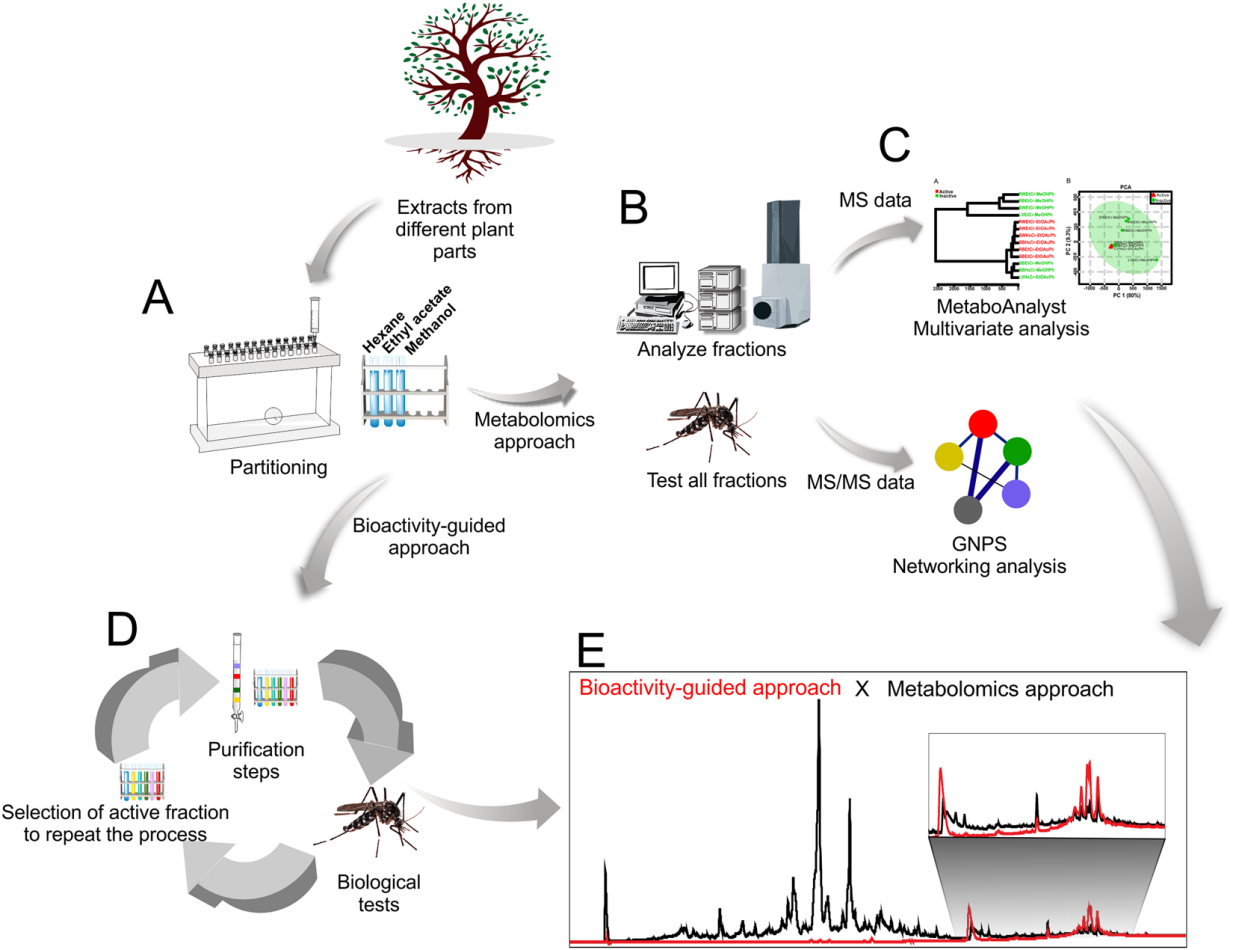
Comparison of the bioactivity-guided approach and metabolomics approach. The first step (**A**) involves, for both approaches, crude extract pre-fractionation. In the metabolomics approach (**B**), all fractions from the crude extracts are submitted to LC-MS/MS analysis and biological testing. The data are analyzed in metabolomics platforms (**C**) indicating the active compounds. (**D**) represents the cycle of purification steps, where fractions are submitted to purification in chromatographic columns and all fractions are tested. After identifying the active fraction, repeated purification steps are performed until the enriched fraction with active compounds is obtained. (**E**) the black chromatogram represents one active fraction where the active compounds are highlighted (RT 28.0–32.0 min), while the red chromatogram, obtained after previous purification steps, indicates the same active compounds previously revealed by the metabolomics approach.

Figure 5 summarizes the bio-guided and metabolomics approaches to identify active compounds. Step A is common for both approaches and involves crude extract pre-fractionation, particularly important for metabolomics in terms of extract clean-up and facilitating unbalanced chemical profiles. However, it does not enable the selection of completely different compounds in the fractions (as shown in Fig. 1), which could be considered a bias in the study, influence platform statistical analysis and lead to misinterpretation. Instead, it is a relevant alternative when several extracts from the same organism are unavailable. In the metabolomics approach (Step B), fractions from the crude extracts are submitted to LC-MS/MS analysis and biological testing. The data were analyzed in the present study using the LC-MS (MetaboAnalyst) and LC-MS/MS (GNPS) platforms (Step C). The annotated Annonaceous acetogenins were the most significant features in the active samples and this can be established without isolation. To confirm the results, bioactivity-guided isolation was performed with the active fraction of one crude extract. Step D represents the purification cycle, where fractions are submitted to purification in chromatographic columns and all resulting fractions tested. The larvicidal fraction is repeatedly purified to obtain an enriched fraction containing active compounds. A comparison between the approaches is shown in Step E - the black chromatogram represents one active fraction where the active compounds are highlighted (RT 29–32 min). The red chromatogram shows the classical purification steps, indicating the same active compounds revealed by the metabolomics approach. Despite some visual indications with the naked eye (Fig. 1), the metabolomics approach with multivariate analysis produced more reliable results. Moreover, the strategy using GNPS facilitates dereplication and the identification process, which can be useful in analyzing a vast number of samples.

Metabolomics have been applied in many different research areas, such as diagnostics^19^, chemosystematics^20^, dereplication^21^, among others^22^. In recent work, Graziani and co-workers adopted a metabolomics approach to identify cytotoxic agents from Fabaceae species^23^. The authors used NMR spectroscopy to facilitate the identification of active compounds in a mixture. In an MS-based approach, the possibility of coupling a chromatographic method with MS analysis provided important information regarding the retention time of active compounds. In addition, the previous separation step reduced peak overlap and, using MS and MS/MS data, enabled annotation of the most significant features through analysis in different metabolomic platforms.

In this contribution, we established a workflow using MS analytical tools to perform metabolomics analysis to annotate active natural product compounds from plants. The classical and metabolomics approaches resulted in identification of the same active compounds, thus highlighting the potential of metabolomics to recognize relevant bioactivity features in complex mixtures. Moreover, the limitations and possibilities of 2 different platforms dealing with MS and MS/MS data were discussed.

## Methods

### General

HPLC grade solvents were purchased from J. T. Baker. Ultrapure water (Millipore, MA, USA) and DIO Spe-ed SPE cartridges (2 g, 6 ml, Applied Separations, Allentown, PA, USA) were used. All extracts were from the *Brazilian Cerrado Biome Plant Extract Bank*, Laboratório de Farmacognosia, Universidade de Brasília.

### HPLC-MS/MS analysis

*Annona crassiflora* extracts (~150 mg): stem wood (ethanolic and hexanic crude extracts; SWEtCr/SWHxCr); leaves (ethanolic and hexanic crude extracts; LVEtCr/LVHxCr); root bark (ethanolic crude extract; RBEtCr); root wood (ethanolic crude extract; RWEtCr), and stem bark (ethanolic and hexanic crude extracts; SBEtCr/SBHxCr) were partitioned using Diol cartridges, with hexane (HxPh), ethyl acetate (EtOAcPh) and methanol (MeOHPh) as mobile phases. The fractions were analyzed in HPLC-MS/MS (Shimadzu LC-6AD coupled with ESI-qTOF, Compact, Bruker). The column used was Supelco Ascentis Express C18 (15 cm × 4.6 mm, 2.7 µm particle size), mobile phases: ultrapure water/methanol with 0.1% formic acid (J. T. Baker), at an oven temperature of 40 °C. The analytical elution method started with 5% organic phase and increased to 100% over 30 min. The column was washed and stabilized for an additional 20 min. The applied flow was 0.6 ml/min with a 20 µl injection volume. Ionization source parameters: capillary voltage 3500 V, nebulizer 5.5 bar, dry gas 10 l/min and source temperature 230 °C.

### Larvicidal tests

The larvicidal tests were performed with *Aedes aegypti* Rockefeller strain. Larvae were obtained from infection-free colonies maintained by the Laboratório de Farmacognosia, Universidade de Brasilia. Colony maintenance is in accordance with World Health Organization guidelines. Samples were tested in quadruplicate in 12-well plates containing 10 L3 larvae, 3 ml of water and 50 µl of sample or negative control (<2% dimethyl sulfoxide). Samples were tested at 25 µg/ml for pure compounds, 125 µg/ml for fractions and 250 µg/ml for crude extracts. The LC_50_ values were determined under the same conditions and the concentrations tested were 100, 75, 50, 25, 10 and 1 µg/ml (GraphPad Prism 7.0 software). Larvae mortality was determined 48 h after treatment. Extracts, fractions and compounds that caused >10% larvae mortality were considered active.

### MetaboAnalyst and GNPS platforms

The data obtained from HPLC-MS/MS analysis were converted to.mzXML format using the MsConvert^24^ software. After conversion, the spectra were processed in MZmine^25^ software using the following modules: mass detection (RT 2.5–35 min, centroid); chromatogram builder (MS level 1; minimum height 1.0 × 10^4^; minimum time span 0.5 min; *m/z* tolerance 10 ppm); deconvolution of the spectra (Algorithm Savitzky-Golay); isotopic peaks grouper (*m/z* tolerance 10 ppm; RT tolerance 0.1 min); duplicate peak filtering; smoothing; data alignment (Join aligner; *m/z* tolerance 10 ppm; RT tolerance 0.5 min); gap-filing (intensity tolerance 20%; *m/z* tolerance 10 ppm, RT tolerance 0.5 min); and peak filtering range (0.00–0.5 min). The data were exported and uploaded to the MetaboAnalyst® platform. The data integrity check was default, data filtering was performed by mean intensity value and normalization performed by Pareto data scaling. The LC-MS/MS converted data files (.mzXML) were uploaded to the GNPS platform, divided into active and inactive samples. Network parameters were default.

### Isolation of active and inactive compounds

*Annona crassiflora* Mart. (Annonaceae) hexanic stem wood crude extract (8.7 g) was partitioned in silica cartridges (174 g) and eluted with 800 ml of hexane (yield 10%), ethyl acetate (yield 21%) and methanol (yield 69%). Ethyl acetate and methanol phases were tested in a larvicidal assay obtaining 92.5% and 0% of mortality at 125 µg/ml, respectively. The ethyl acetate phase was purified in a chromatographic column using silica gel and eluted with ethyl acetate. After the collection of 16 fractions (160 ml), the mobile phase proportion was modified, with an increasing methanol gradient. Fractions were analyzed using a Waters HPLC and a C_18_ column (Sunfire 4.6 × 150 mm) used for chromatographic separation. The applied flow was 1 ml/min with a 15 µl injection. The mobile phase was ultrapure water (solvent A) and methanol (solvent B), both with 0.1% (v/v) formic acid. The gradient elution method started with 20% B, increased to 60% B until 2 min and to 80% until 10 min. A soft gradient was applied from 80–85% until 80 min. The column was washed and stabilized for an additional 10 min.

Fractions were grouped as follows: CCC1_Fr1 (7.4%); CCC1_Fr2 (0.8%); CCC1_Fr3 (1.0%); CCC1_Fr4 (1.6%); CCC1_Fr5 (10.1%); CCC1_Fr6 (5.4%), and CCC1_Fr7 (73.7%). The last fraction proved the most active, causing 92.5% mortality, and was further chromatographed (996 mg) in a Sephadex LH-20 column with methanol as mobile phase. The resulting 6 fractions were subsequently analysed by HPLC: CCC2_Fr1 (36%); CCC2_Fr2 (22%); CCC2_Fr3 (1.5%); CCC2_Fr4 (1.3%); CCC2_Fr5 (1.2%); CCC2_Fr6 (0.8%). Fractions CCC2_Fr2 and CCC2_Fr3 showed 97.5% and 80% activity, respectively. CCC2_Fr2 was submitted to HPLC-preparative C18 column Varian Dynamex (250 × 21.4 mm). The gradient elution method started with 20% B and increased to 90% B until 80 min, with 20 ml/min flow. The column was washed and stabilized for an additional 10 min. Finally, 4 fractions were obtained after prep HPLC - Prep_Fr1 to Prep_Fr4 were submitted for structural elucidation.

## Supplementary information

Supporting Information.
