# Supplementary material for: Mass spectrometry-based metabolomics approach in the isolation of bioactive natural products
Source: Sci Rep. 2020 Jan 23;10:1051. doi: 10.1038/s41598-020-58046-y (PMC6978511; doi:10.1038/s41598-020-58046-y)
Supplement: Supplementary file 1 — Supporting Information. [file 41598_2020_58046_MOESM1_ESM.docx]

**Mass spectrometry-based metabolomics approach in the isolation of bioactive natural products**

**Daniel P. Demarque^1^, Renata G. Dusi^1^, Francisco D. M. de Sousa^1^, Sophia M. Grossi^1^, Maira R. S. Silvério^2^, Norberto P. Lopes^2^, Laila S. Espindola^1^*.**

^1^ Laboratório de Farmacognosia, Universidade de Brasília, Brasília, Brazil

^2^ Núcleo de Pesquisa em Produtos Naturais e Sintéticos, Departamento de Física e Química, Faculdade de Ciências Farmacêuticas de Ribeirão Preto, Universidade de São Paulo, Brazil.

Correspondence: Prof. Dr. Laila Salmen Espindola, Laboratório de Farmacognosia, Universidade de Brasília, Campus Universitário Darcy Ribeiro, Asa Norte, Postal Code 70910-900, Brasília, DF, Brazil. Phone: + 55 61 3107 2016; Fax: + 55 61 3107 1943 darvenne@unb.br

**INDEX**

[Table S1: Yield from partitioning procedures using diol cartridges and hexane (HxPh), ethyl acetate (EtOAcPh) and methanol (MeOHPh) as mobile phases. 2](#_Toc23230749)

[Table S2: *Aedes aegypti* (Rockefeller strain) larvae mortality with *A. crassiflora* extracts. 3](#_Toc23230750)

[Figure S1: Molecular networking obtained in the GNPS platform. 4](#_Toc23230697)

[Figure S2: Isolated compounds in mixture obtained after classical bioguided approach (HPLC-prep fractions). 5](#_Toc23230698)

[Figure S3: Direct-infusion MS spectra of fraction Prep_Fr1 obtained by bioactivity-guided isolation. 5](#_Toc23230699)

[Figure S4: MS/MS spectra of 661.46 from fraction Prep_Fr1. 6](#_Toc23230700)

[Figure S5: Direct-infusion MS spectra of fraction Prep_Fr2 obtained by bioactivity-guided isolation. 6](#_Toc23230701)

[Figure S6: MS/MS spectra of 635.45 from fraction Prep_Fr2. 7](#_Toc23230702)

[Figure S7: MS/MS spectra of 679.47 from fraction Prep_Fr2. 7](#_Toc23230703)

[Figure S8: MS/MS spectra of 661.46 from fraction Prep_Fr2. 8](#_Toc23230704)

[Figure S9: Direct-infusion MS spectra of fraction Prep_Fr3 obtained by bioactivity-guided isolation. 8](#_Toc23230705)

[Figure S10: MS/MS spectra of 617.44 from fraction Prep_Fr3. 9](#_Toc23230706)

[Figure S11: MS/MS spectra of 635.45 from fraction Prep_Fr3. 9](#_Toc23230707)

[Figure S12: MS/MS spectra of 705.49 from fraction Prep_Fr3. 10](#_Toc23230708)

[Figure S13: MS/MS spectra of 645.47 from fraction Prep_Fr3. 10](#_Toc23230709)

[Figure S14: MS/MS spectra of 663.48 from fraction Prep_Fr3. 11](#_Toc23230710)

[Figure S15: Direct-infusion MS spectra of fraction Prep_Fr4 obtained by bioactivity-guided isolation. 11](#_Toc23230711)

[Figure S16: Prep_FR1 ^1^H NMR. 12](#_Toc23230712)

[Figure S17: Prep_FR1 ^13^C NMR. 12](#_Toc23230713)

[Figure S18: Prep_FR1 COSY NMR. 13](#_Toc23230714)

[Figure S19: Prep_FR1 HSQC NMR. 13](#_Toc23230715)

[Figure S20: Prep_FR1 HMBC NMR. 14](#_Toc23230716)

[Figure S21: Prep_FR2 ^1^H NMR. 14](#_Toc23230717)

[Figure S22: Prep_FR2 ^13^C NMR. 15](#_Toc23230718)

[Figure S23: Prep_FR2 COSY NMR. 15](#_Toc23230719)

[Figure S24: Prep_FR2 HSQC NMR. 16](#_Toc23230720)

[Figure S25: Prep_FR2 HMBC NMR. 16](#_Toc23230721)

[Figure S26: Prep_FR3 ^1^H NMR. 17](#_Toc23230722)

[Figure S27: Prep_FR3 COSY NMR. 17](#_Toc23230723)

[Figure S28: Prep_FR3 HSQC NMR. 18](#_Toc23230724)

[Figure S29: Prep_FR3 HMBC NMR. 18](#_Toc23230725)

[Figure S30: Prep_FR4 ^1^H NMR. 19](#_Toc23230726)

[Figure S31: Prep_FR4 ^13^C NMR. 19](#_Toc23230727)

[Figure S32: Prep_FR4 COSY NMR. 20](#_Toc23230728)

[Figure S33: Prep_FR4 HSQC NMR. 20](#_Toc23230729)

[Figure S34: Prep_FR4 HMBC NMR. 21](#_Toc23230730)

[Figure S35: Mortality versus log 10 concentration of fraction Prep_Fr1 (LC_50_= 10.4 µg/ml). 21](#_Toc23230731)

[Figure S36: Mortality versus log 10 concentration of fraction Prep_Fr2 (LC_50_= 9.0 µg/ml). 22](#_Toc23230732)

[Figure S37: Mortality versus log 10 concentration of fraction Prep_Fr3 (LC_50_= 3.7 µg/ml). 22](#_Toc23230733)

[Figure S38: Mortality versus log 10 concentration of fraction Prep_Fr4 (LC_50_= 6.4 µg/ml). 22](#_Toc23230734)

Table S1: Yield from partitioning procedures using diol cartridges and hexane (HxPh), ethyl acetate (EtOAcPh) and methanol (MeOHPh) as mobile phases.

|  | Crude extract | Partition solvent | Yield (%) | Code |
| --- | --- | --- | --- | --- |
| 1 | SWEtCr | Hexane | 1.0 | SWEtCr-HxPh |
|  |  | Ethyl acetate | 35.8 | SWEtCr-EtOAcPh |
|  |  | Methanol | 47.2 | SWEtCr-MeOHPh |
| 2 | SWHxCr | Hexane | 31.9 | SWHxCr-HxPh |
|  |  | Ethyl acetate | 46.1 | SWHxCr-EtOAcPh |
|  |  | Methanol | 0.0 | SWHxCr-MeOHPh |
| 3 | LVEtCr | Hexane | 1.6 | LVEtCr-HxPh |
|  |  | Ethyl acetate | 8.6 | LVEtCr-EtOAcPh |
|  |  | Methanol | 64.5 | LVEtCr-MeOHPh |
| 4 | LVHxCr | Hexane | 31.1 | LVHxCr-HxPh |
|  |  | Ethyl acetate | 62.8 | LVHxCr-EtOAcPh |
|  |  | Methanol | 3.9 | LVHxCr-MeOHPh |
| 5 | RBEtCr | Hexane | 1.2 | RBEtCr-HxPh |
|  |  | Ethyl acetate | 29.4 | RBEtCr-EtOAcPh |
|  |  | Methanol | 44.5 | RBEtCr-MeOHPh |
| 6 | RWEtCr | Hexane | 1.4 | RWEtCr-HxPh |
|  |  | Ethyl acetate | 37.4 | RWEtCr-EtOAcPh |
|  |  | Methanol | 43.1 | RWEtCr-MeOHPh |
| 7 | SBHxCr | Hexane | 9.5 | SBHxCr-HxPh |
|  |  | Ethyl acetate | 25.7 | SBHxCr-EtOAcPh |
|  |  | Methanol | 19.3 | SBHxCr-MeOHPh |
| 8 | SBEtCr | Hexane | 1.1 | SBEtCr-HxPh |
|  |  | Ethyl acetate | 32.9 | SBEtCr-EtOAcPh |
|  |  | Methanol | 36.9 | SBEtCr-MeOHPh |

Crude extracts from *A. crassiflora*: -

SWEtCr: Stem wood ethanolic crude extract

SWHxCr: Stem wood hexanic crude extract

LVEtCr: Leaves ethanolic crude extract

LVHxCr: Leaves hexanic crude extract

RBEtCr: Root bark ethanolic crude extract

RWEtCr: Root wood ethanolic crude extract

SBEtCr: Stem bark ethanolic crude extract

SBHxCr: Stem bark hexanic crude extract

Table S2: *Aedes aegypti* (Rockefeller strain) larvae mortality with *A. crassiflora* extracts.

| Extract | Rep1 | Rep2 | Rep3 | Rep4 | Average | Mortality (%) |
| --- | --- | --- | --- | --- | --- | --- |
| SWEtCr-EtOAcPh | 0 | 5 | 10 | 3 | 4.5 | 45 |
| SWHxCr-EtOAcPh | 0 | 1 | 2 | 2 | 1.25 | 12.5 |
| LVHXCr-EtOAcPh | 0 | 0 | 0 | 0 | 0 | 0 |
| RBEtCr-EtOAcPh | 8 | 5 | 9 | 6 | 7 | 70 |
| RWEtCr-EtOAcPh | 4 | 6 | 5 | 5 | 5 | 50 |
| SBHxCr-EtOAcPh | 3 | 3 | 6 | 2 | 3.5 | 35 |
| SBEtCr-EtOAcPh | 6 | 1 | 1 | 5 | 3.25 | 32.5 |
| SWEtCr-MeOHPh | 0 | 0 | 0 | 0 | 0 | 0 |
| LVEtCr-MeOHPh | 0 | 0 | 0 | 0 | 0 | 0 |
| RBEtCr-MeOHPh | 0 | 0 | 0 | 0 | 0 | 0 |
| RWEtCr-MeOHPh | 1 | 0 | 0 | 0 | 0.25 | 2.5 |
| SBHxCr-MeOHPh | 0 | 0 | 0 | 0 | 0 | 0 |
| SBEtCr-MeOHPh | 0 | 0 | 0 | 0 | 0 | 0 |

*Rep: replicates; Larvae mortality was determined after 48 h exposure.

Crude extracts from *A. crassiflora*:

SWEtCr: Stem wood ethanolic crude extract

SWHxCr: Stem wood hexanic crude extract

LVEtCr: Leaves ethanolic crude extract

LVHxCr: Leaves hexanic crude extract

RBEtCr: Root bark ethanolic crude extract

RWEtCr: Root wood ethanolic crude extract

SBEtCr: Stem bark ethanolic crude extract

SBHxCr: Stem bark hexanic crude extract

EtOAcPh: Ethyl acetate phase

MeOHPh: Methanolic phase


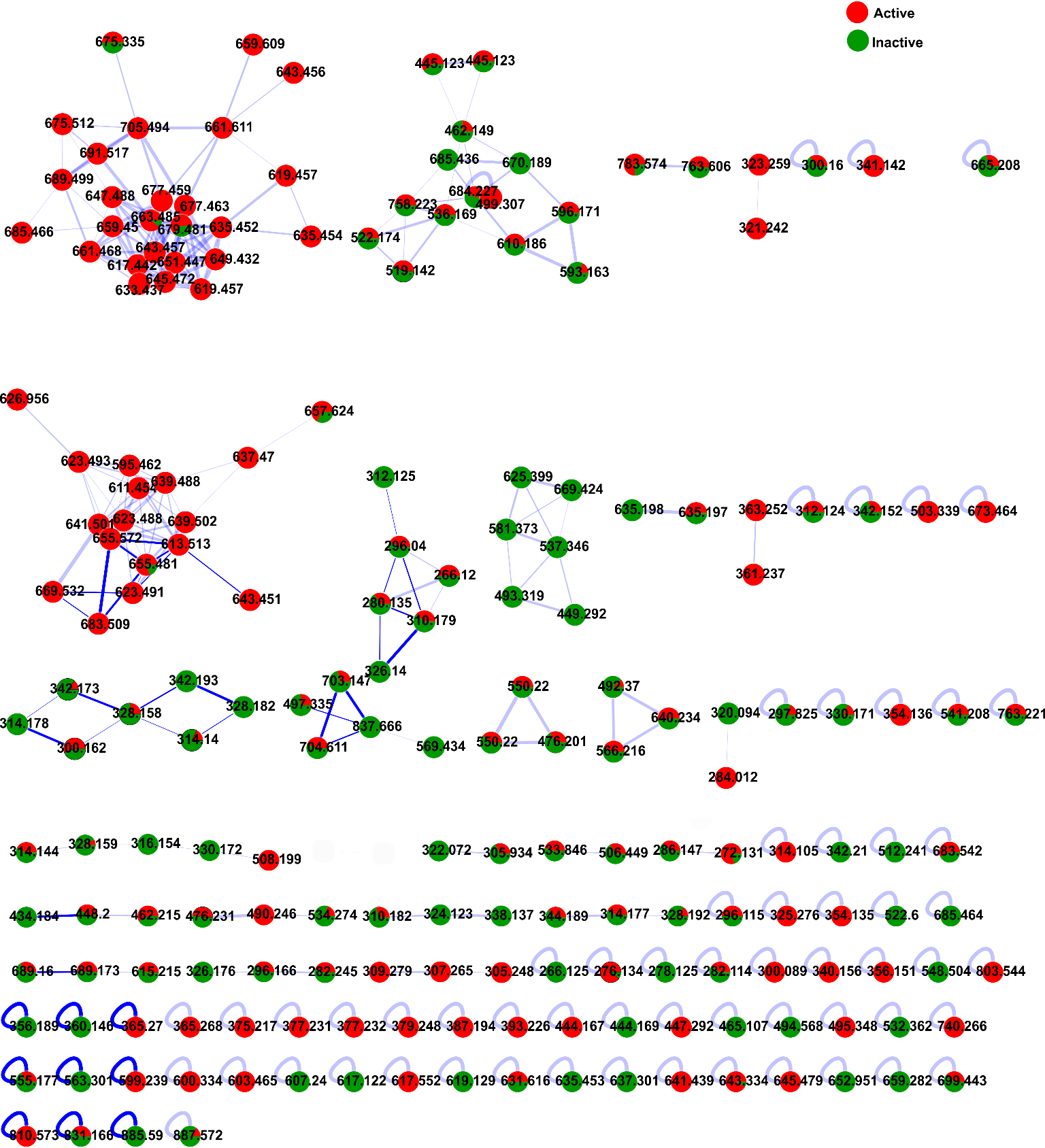


Figure S1: Molecular networking obtained in the GNPS platform.


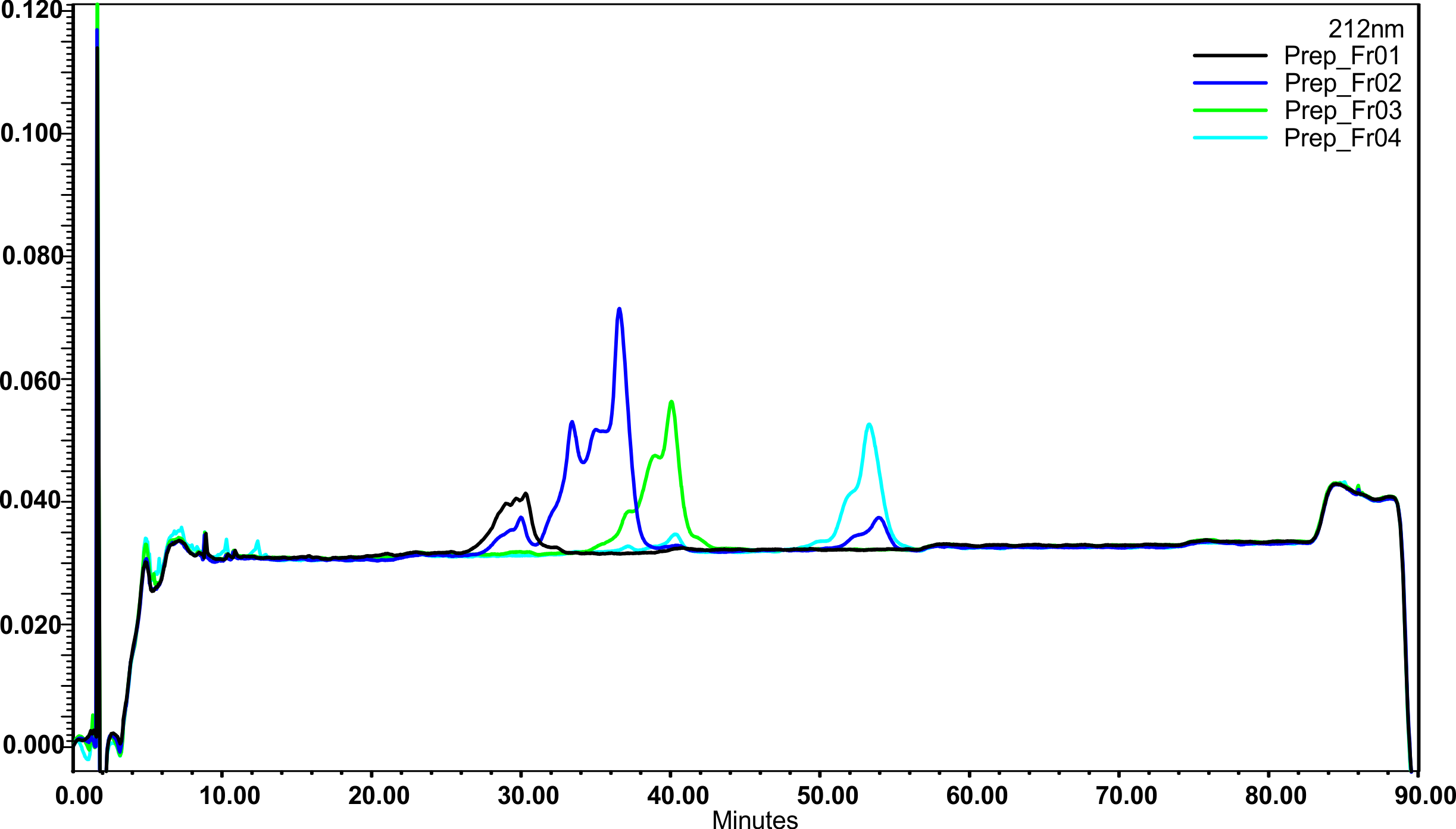


**AU**

Figure S2: Isolated compounds in mixture obtained after classical bioguided approach (HPLC-prep fractions).


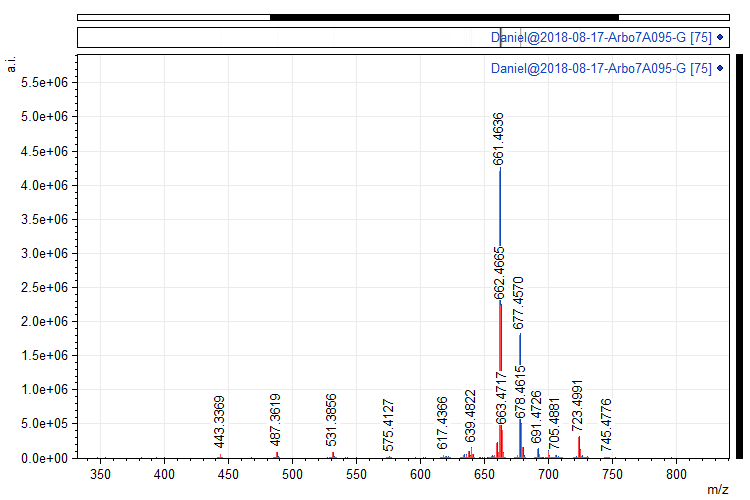


**Figure S3:** Direct-infusion MS spectra of fraction Prep_Fr1 obtained by bioactivity-guided isolation.


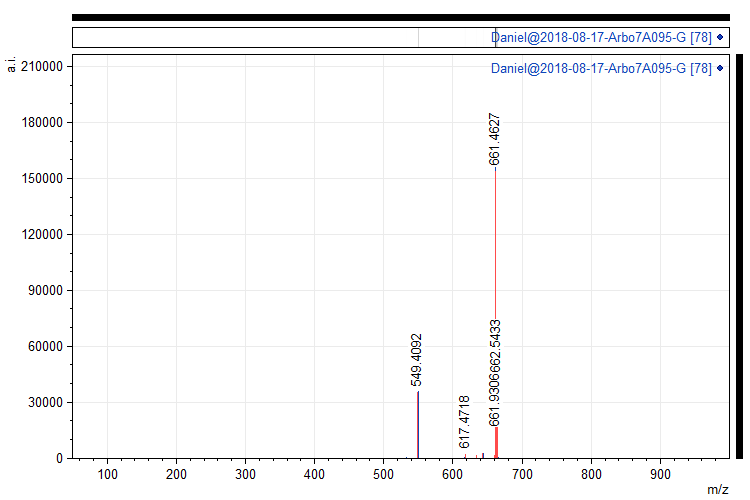


Figure S4: MS/MS spectra of 661.46 from fraction Prep_Fr1.


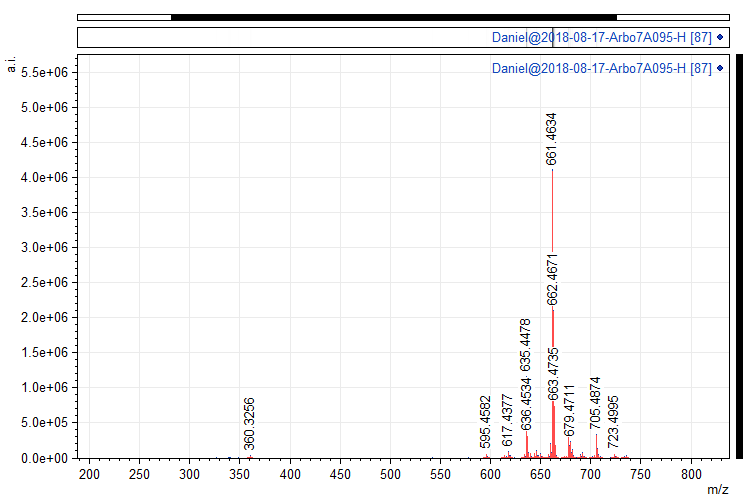


Figure S5: Direct-infusion MS spectra of fraction Prep_Fr2 obtained by bioactivity-guided isolation.


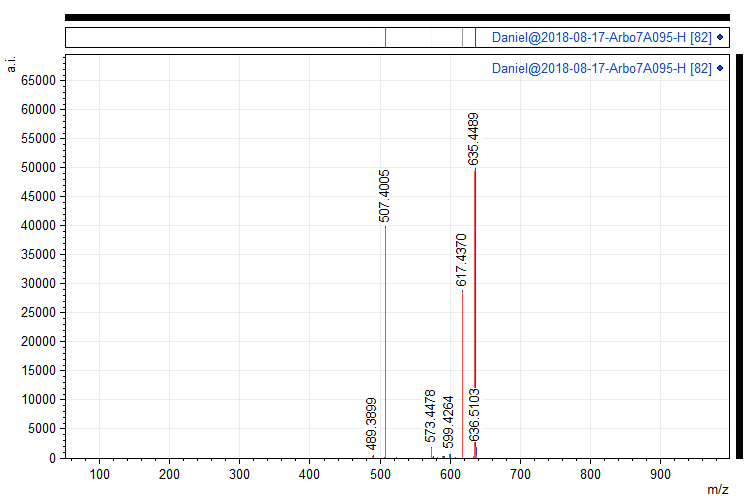


Figure S6: MS/MS spectra of 635.45 from fraction Prep_Fr2.


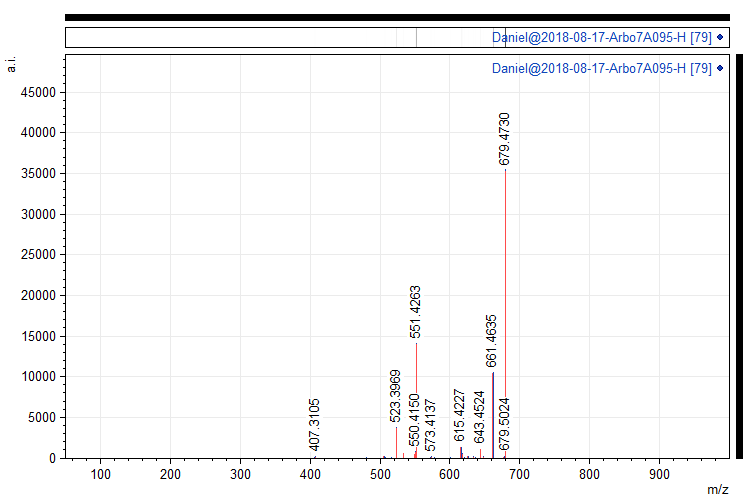


Figure S7: MS/MS spectra of 679.47 from fraction Prep_Fr2.


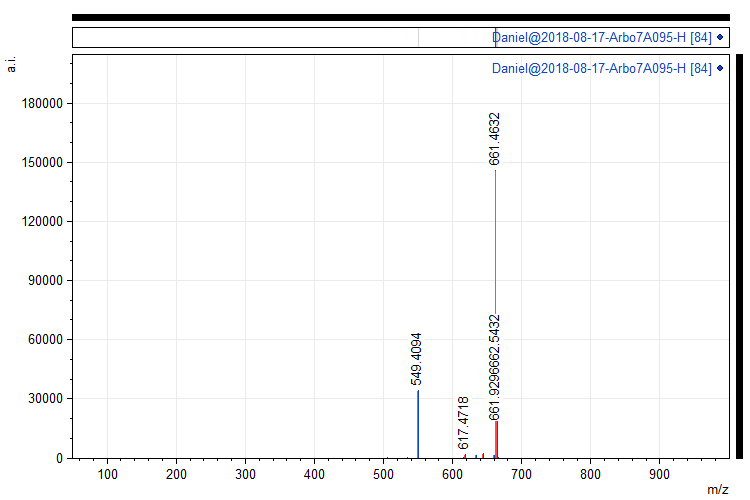


Figure S8: MS/MS spectra of 661.46 from fraction Prep_Fr2.


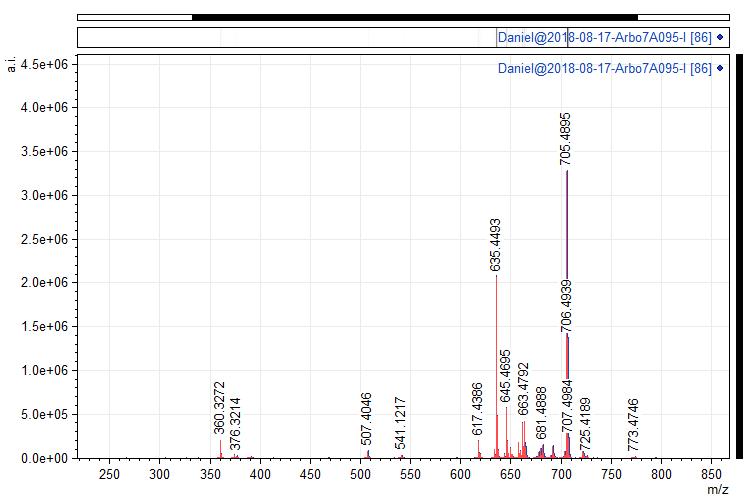


Figure S9: Direct-infusion MS spectra of fraction Prep_Fr3 obtained by bioactivity-guided isolation.


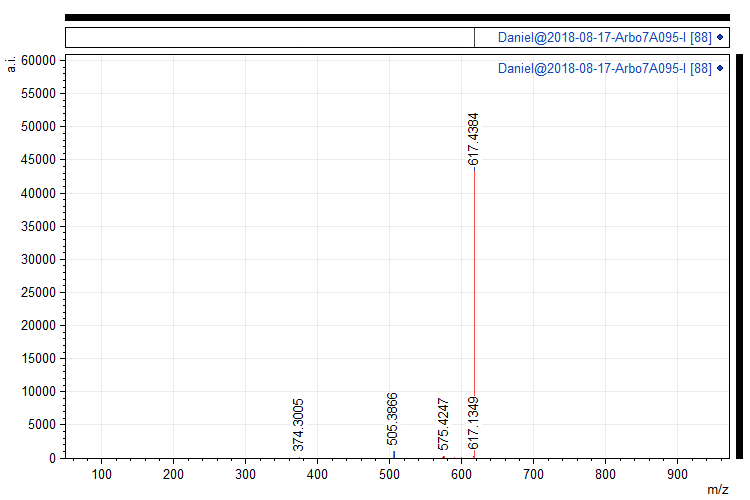


Figure S10: MS/MS spectra of 617.44 from fraction Prep_Fr3.


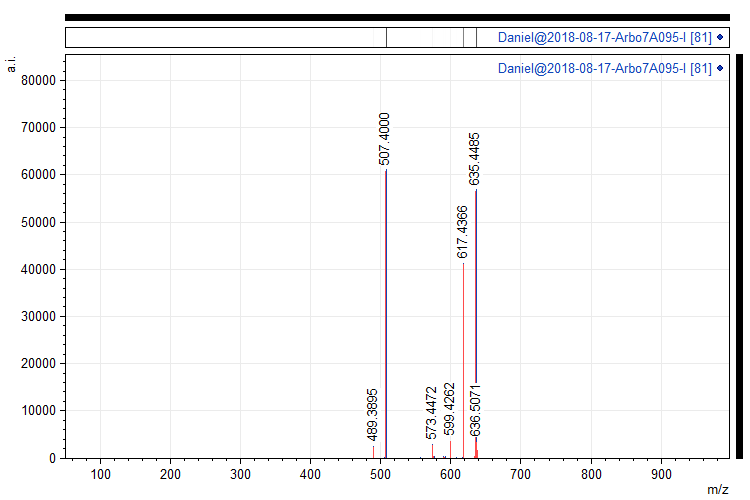


Figure S11: MS/MS spectra of 635.45 from fraction Prep_Fr3.


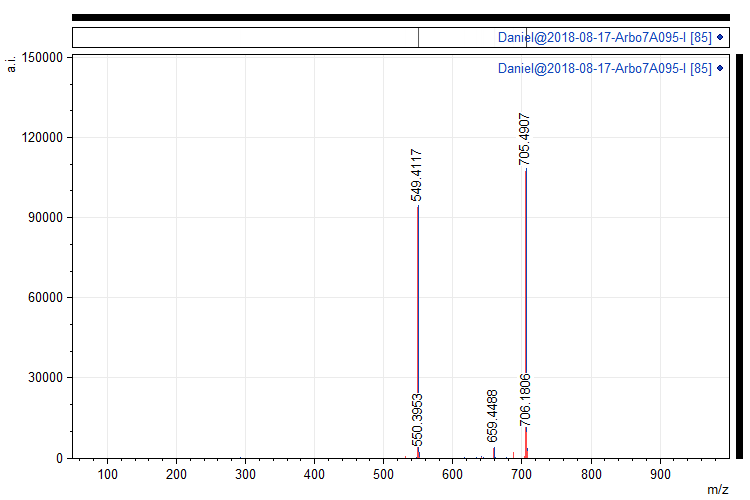


Figure S12: MS/MS spectra of 705.49 from fraction Prep_Fr3.


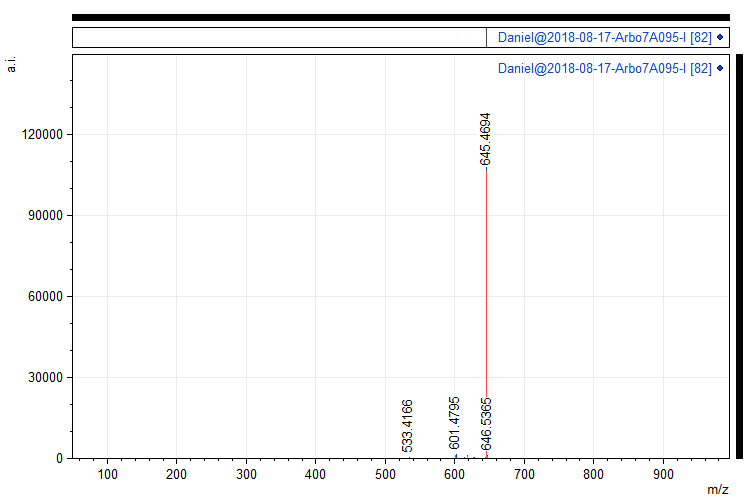


Figure S13: MS/MS spectra of 645.47 from fraction Prep_Fr3.


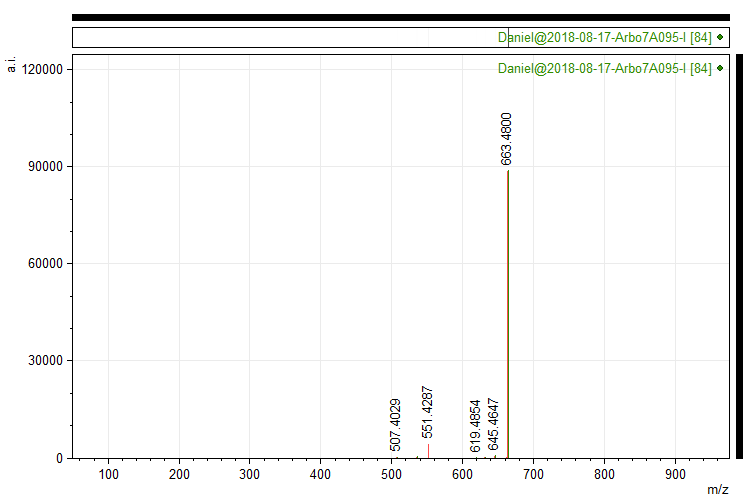


Figure S14: MS/MS spectra of 663.48 from fraction Prep_Fr3.


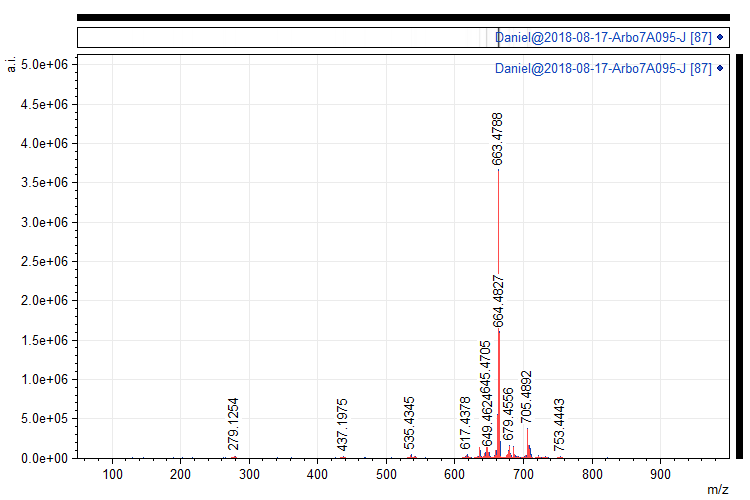


Figure S15: Direct-infusion MS spectra of fraction Prep_Fr4 obtained by bioactivity-guided isolation.


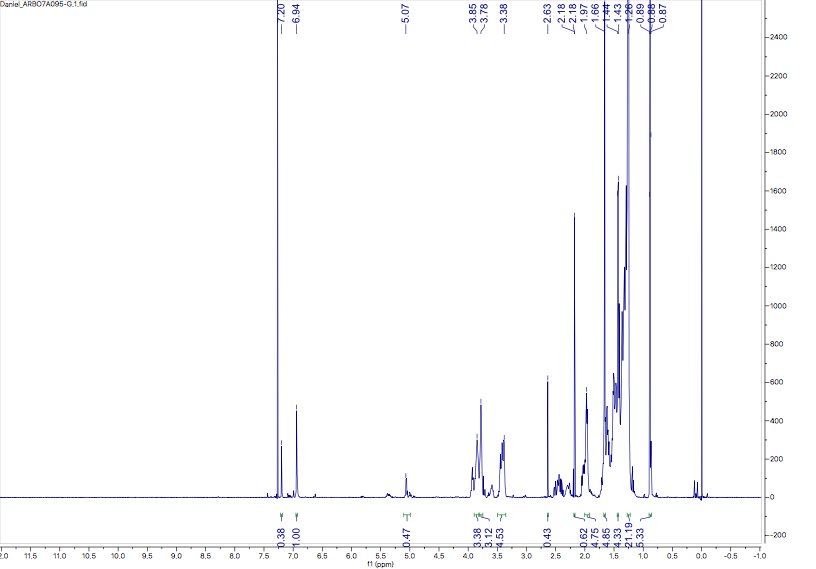


Figure S16: Prep_FR1 ^1^H NMR.


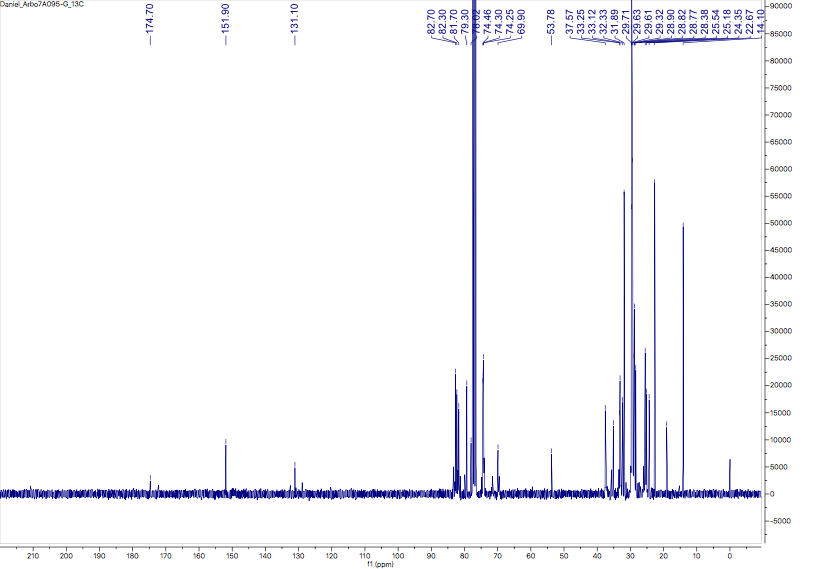


Figure S17: Prep_FR1 ^13^C NMR.


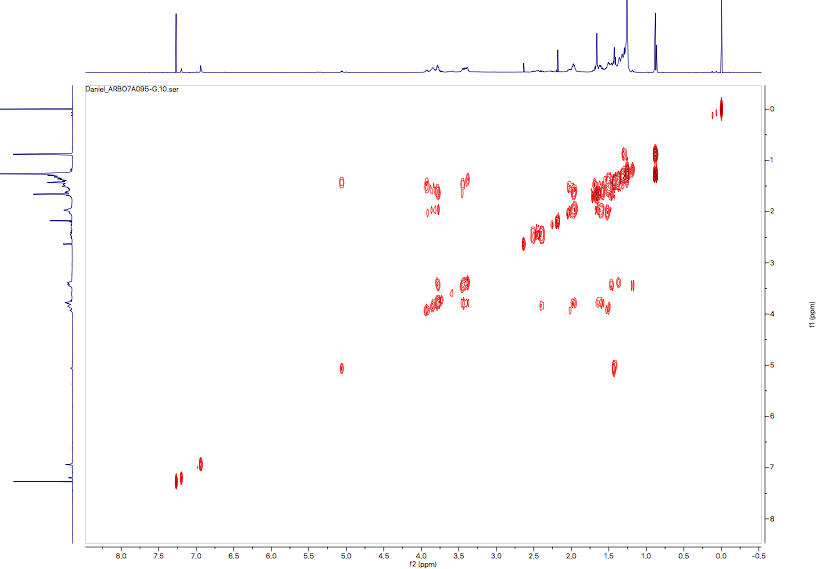


Figure S18: Prep_FR1 COSY NMR.


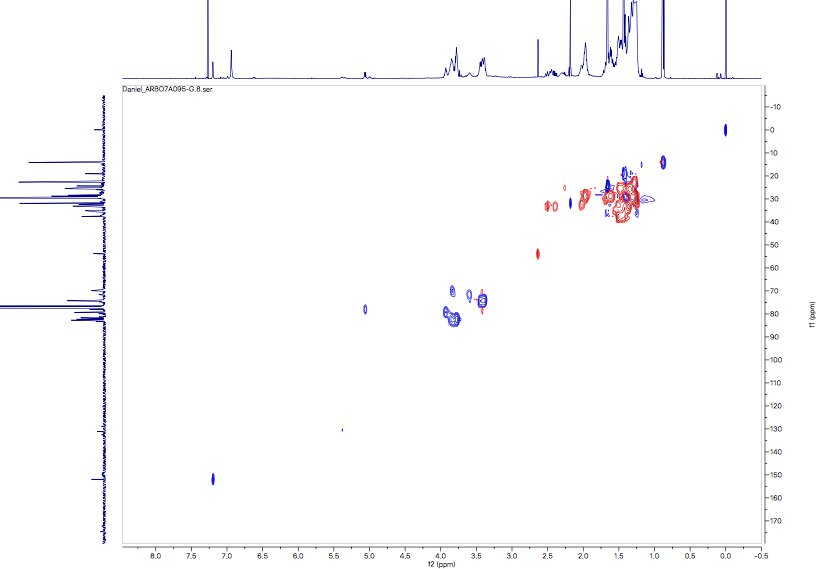


Figure S19: Prep_FR1 HSQC NMR.


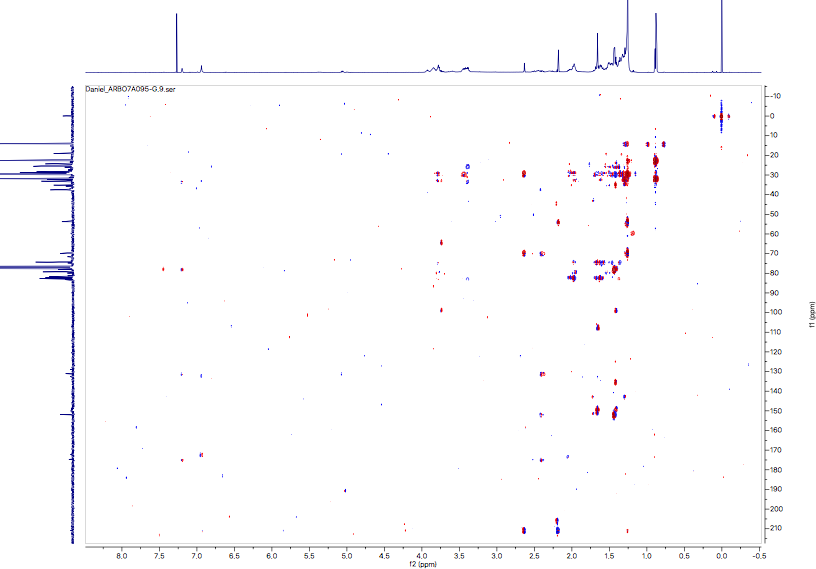


Figure S20: Prep_FR1 HMBC NMR.


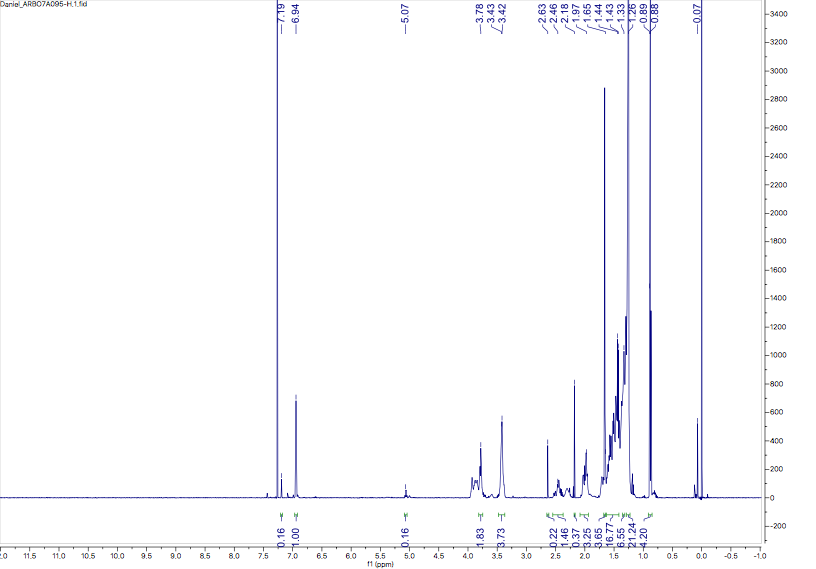


Figure S21: Prep_FR2 ^1^H NMR.


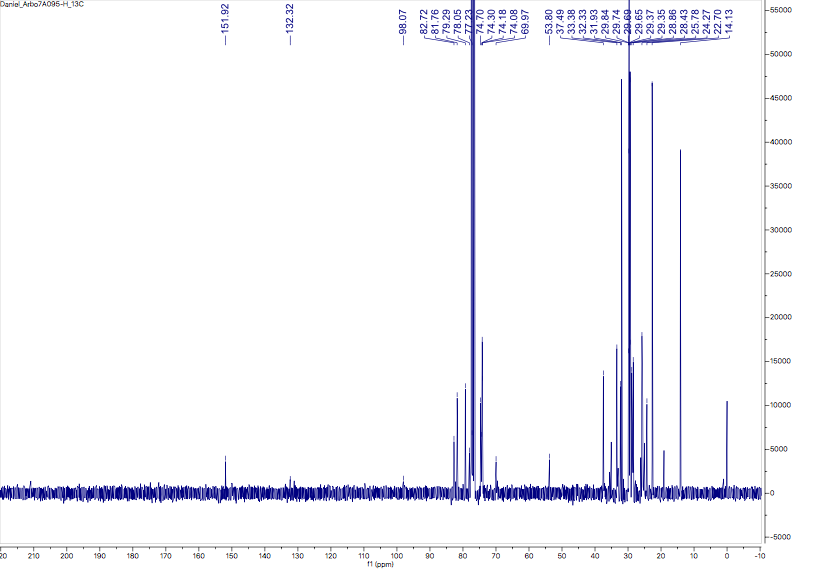


Figure S22: Prep_FR2 ^13^C NMR.


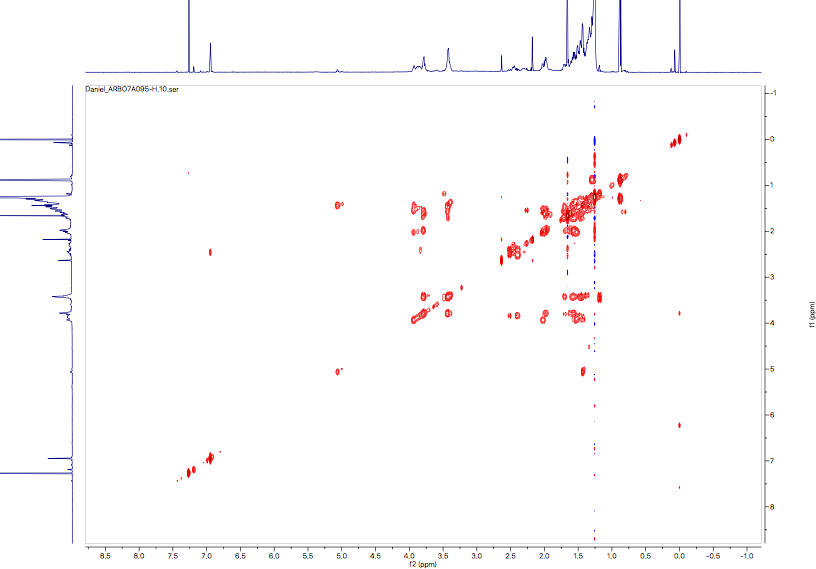


Figure S23: Prep_FR2 COSY NMR.


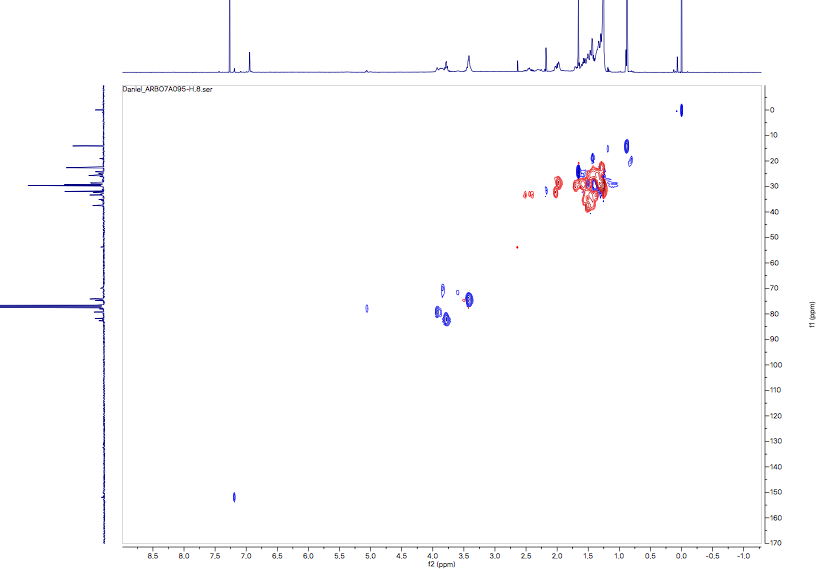


Figure S24: Prep_FR2 HSQC NMR.


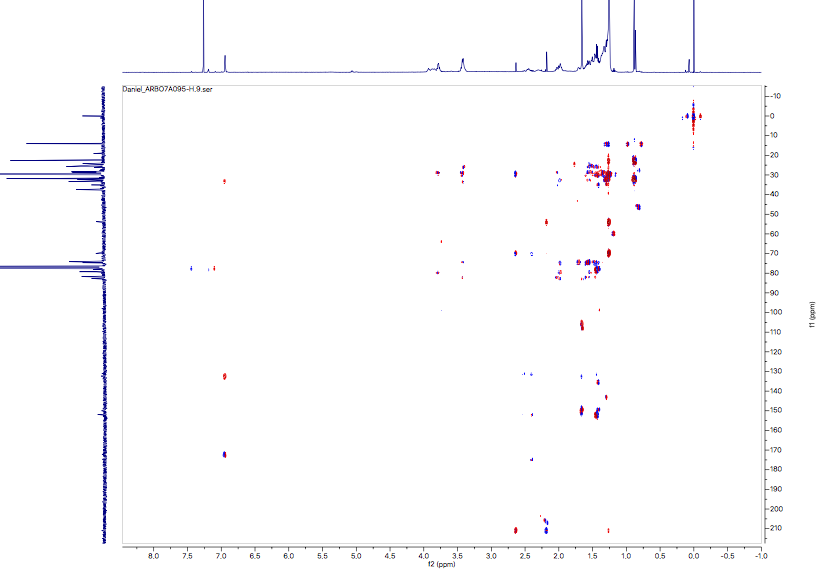


Figure S25: Prep_FR2 HMBC NMR.


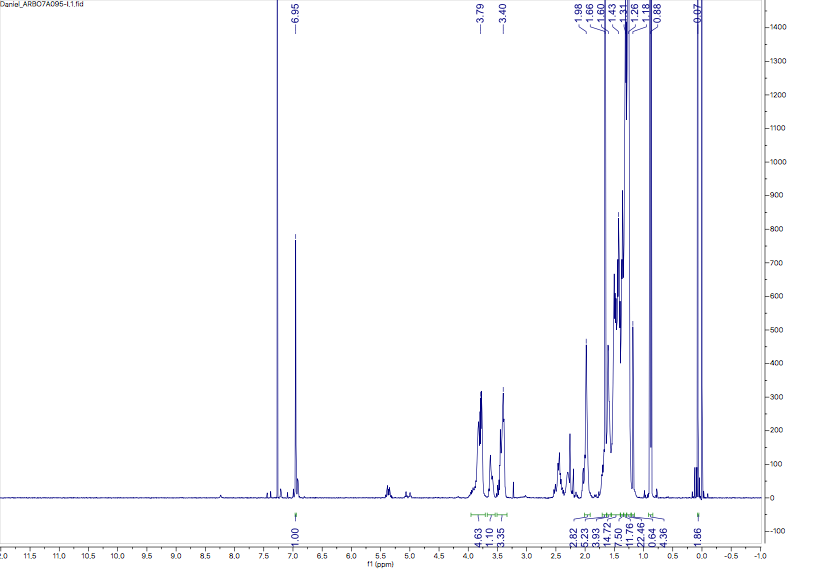


Figure S26: Prep_FR3 ^1^H NMR.


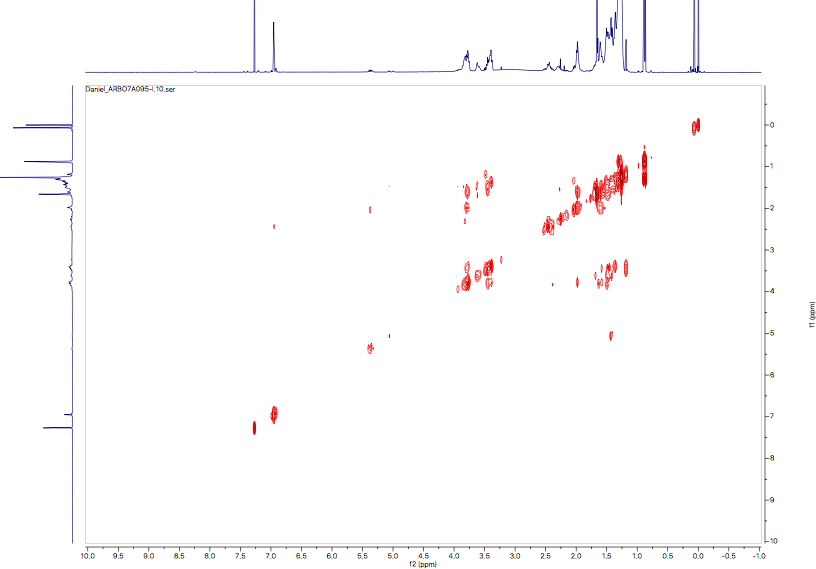


Figure S27: Prep_FR3 COSY NMR.


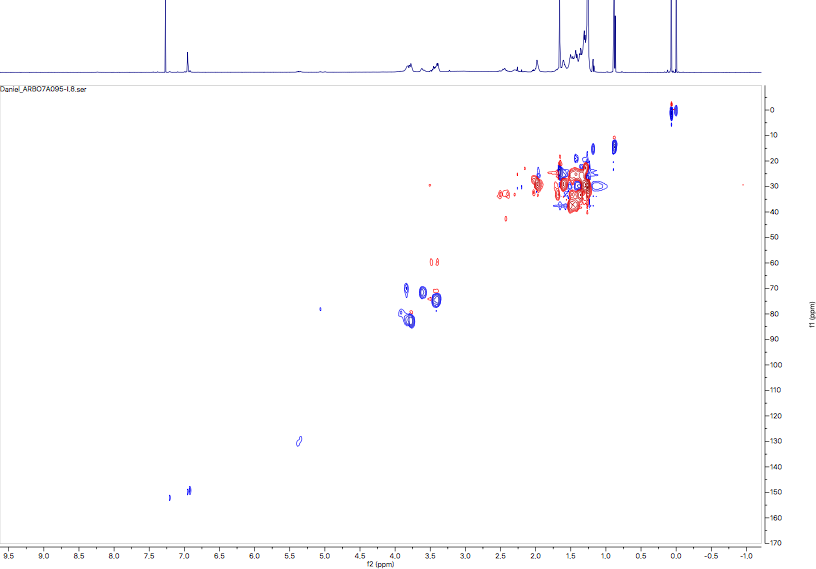


Figure S28: Prep_FR3 HSQC NMR.


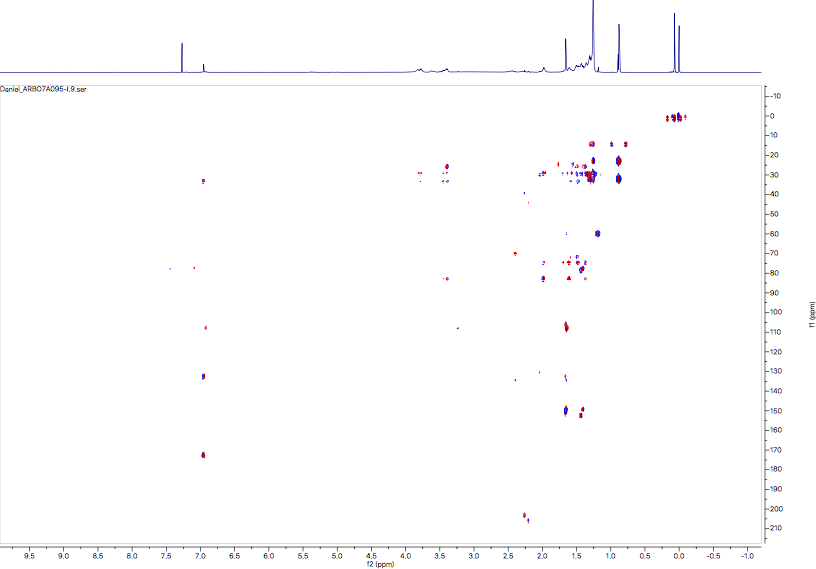


Figure S29: Prep_FR3 HMBC NMR.


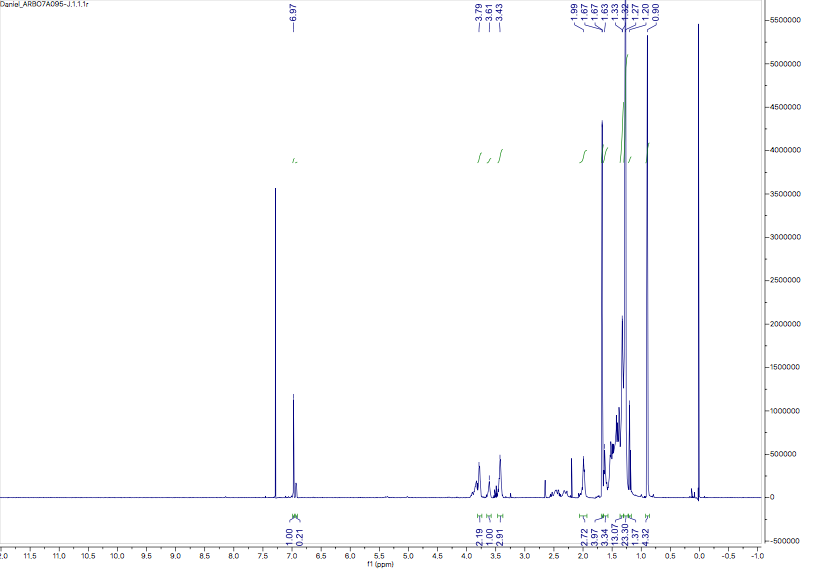


Figure S30: Prep_FR4 ^1^H NMR.


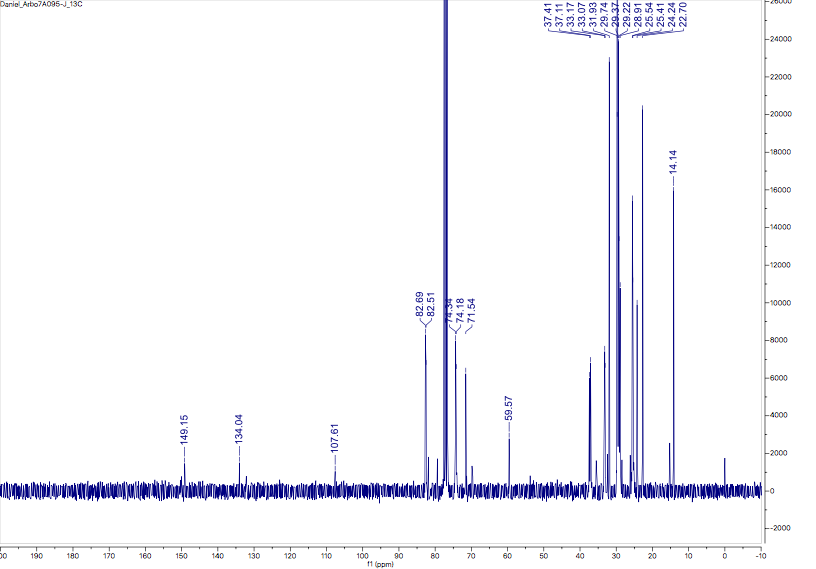


Figure S31: Prep_FR4 ^13^C NMR.


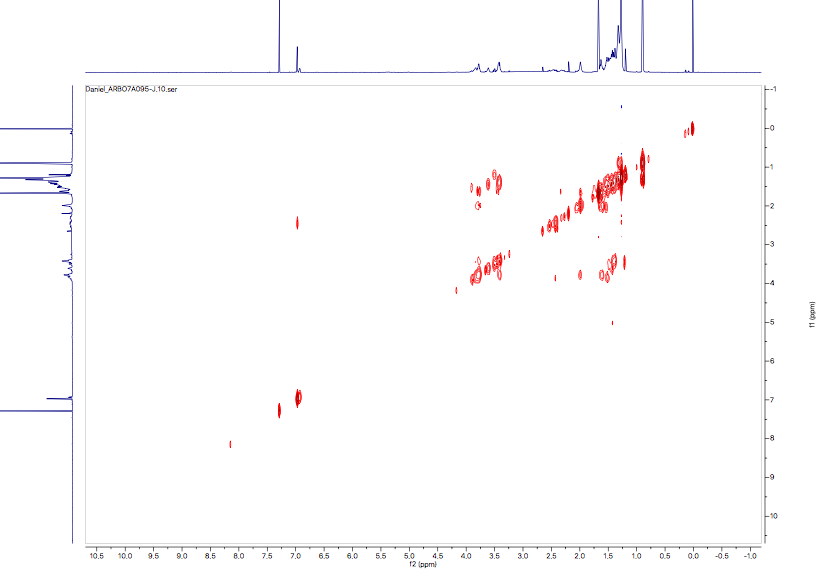


Figure S32: Prep_FR4 COSY NMR.


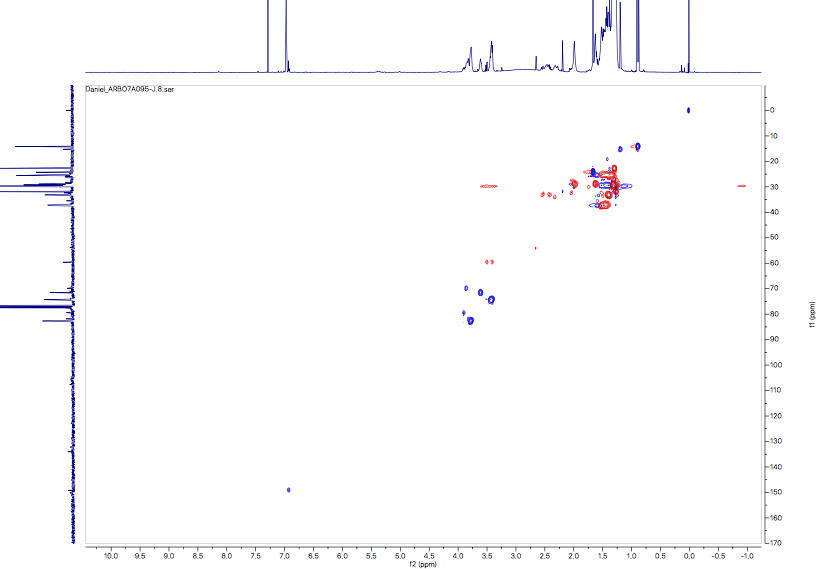


Figure S33: Prep_FR4 HSQC NMR.


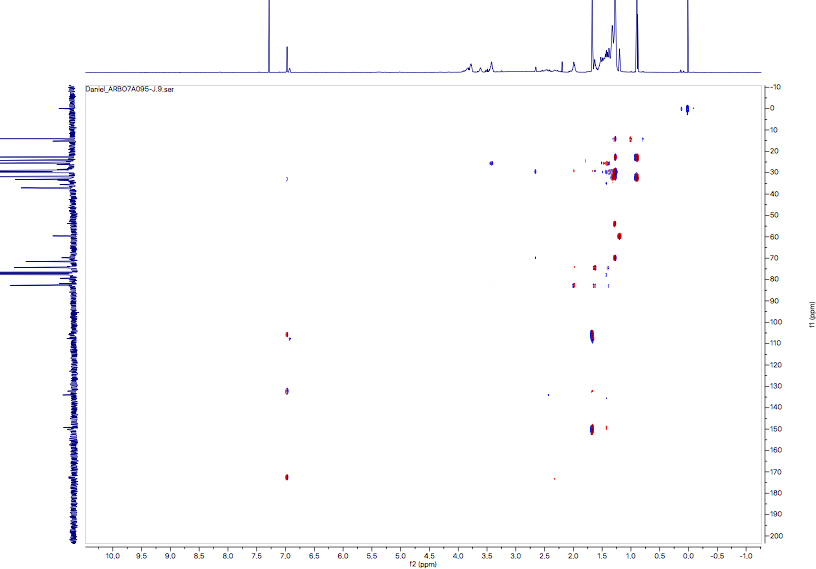


Figure S34: Prep_FR4 HMBC NMR.


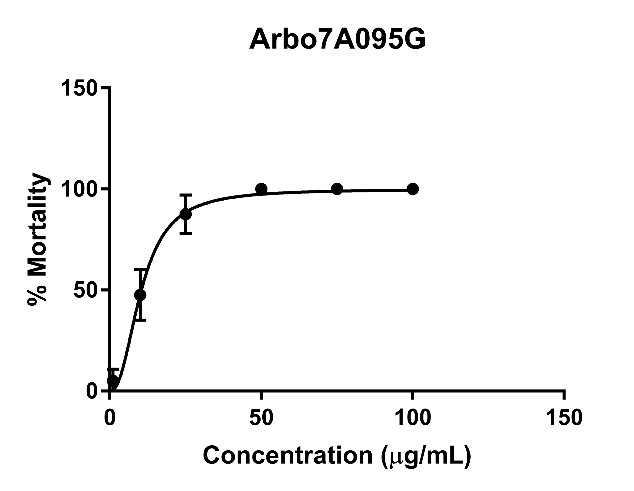


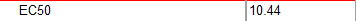


Figure S35: Mortality versus log 10 concentration of fraction Prep_Fr1 (LC_50_= 10.4 µg/ml).


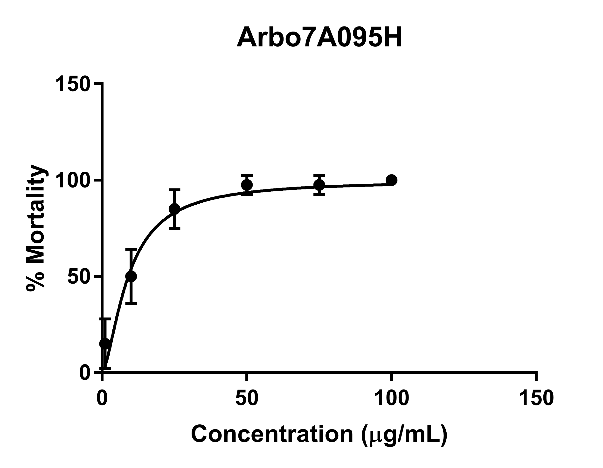


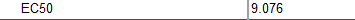


Figure S36: Mortality versus log 10 concentration of fraction Prep_Fr2 (LC_50_= 9.0 µg/ml).


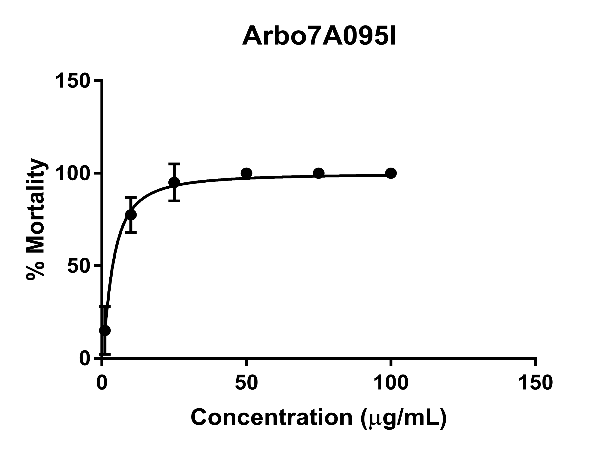


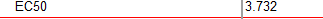


Figure S37: Mortality versus log 10 concentration of fraction Prep_Fr3 (LC_50_= 3.7 µg/ml).


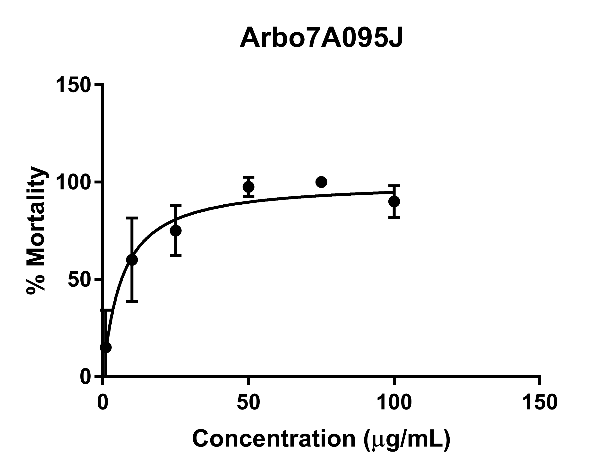


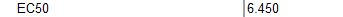


Figure S38: Mortality versus log 10 concentration of fraction Prep_Fr4 (LC_50_= 6.4 µg/ml).
